# Source cell-type epigenetic memory persists in induced pluripotent cells but is lost in subsequently derived germline cells

**DOI:** 10.3389/fcell.2024.1306530

**Published:** 2024-02-12

**Authors:** Yu-Huey Lin, Jake D. Lehle, John R. McCarrey

**Affiliations:** Department of Neuroscience, Developmental and Regenerative Biology, The University of Texas at San Antonio, San Antonio, TX, United States

**Keywords:** epigenetics, reprogramming, transcriptional memory, stem cell therapy, *in vitro* gametogenesis

## Abstract

**Introduction:** Retention of source cell-type epigenetic memory may mitigate the potential for induced pluripotent stem cells (iPSCs) to fully achieve transitions in cell fate *in vitro*. While this may not preclude the use of iPSC-derived somatic cell types for therapeutic applications, it becomes a major concern impacting the potential use of iPSC-derived germline cell types for reproductive applications. The transition from a source somatic cell type to iPSCs and then on to germ-cell like cells (GCLCs) recapitulates two major epigenetic reprogramming events that normally occur during development *in vivo*—embryonic reprogramming in the epiblast and germline reprogramming in primordial germ cells (PGCs). We examined the extent of epigenetic and transcriptomic memory persisting first during the transition from differentiated source cell types to iPSCs, and then during the transition from iPSCs to PGC-like cells (PGCLCs).

**Methods:** We derived iPSCs from four differentiated mouse cell types including two somatic and two germ cell types and tested the extent to which each resulting iPSC line resembled a) a validated ES cell reference line, and b) their respective source cell types, on the basis of genome-wide gene expression and DNA methylation patterns. We then induced each iPSC line to form PGCLCs, and assessed epigenomic and transcriptomic memory in each compared to endogenous PGCs/M-prospermatogonia.

**Results:** In each iPSC line, we found residual gene expression and epigenetic programming patterns characteristic of the corresponding source differentiated cell type from which each was derived. However, upon deriving PGCLCs, we found very little evidence of lingering epigenetic or transcriptomic memory of the original source cell type.

**Discussion:** This result indicates that derivation of iPSCs and then GCLCs from differentiated source cell types *in vitro* recapitulates the two-phase epigenetic reprogramming that normally occurs *in vivo*, and that, to a significant extent, germline cell types derived *in vitro* from pluripotent cells accurately recapitulate epigenetic programming and gene expression patterns corresponding to equivalent endogenous germ cell types, suggesting that they have the potential to form the basis of *in vitro* gametogenesis as a useful therapeutic strategy for treatment of infertility.

## Introduction

Pluripotent stem cells (PSCs), including embryonic stem cells (ESCs) and induced pluripotent stem cells (iPSCs), have the ability to self-renew or initiate differentiation into any cell type in the body, including germ cells (Yamanaka, 2007; Yamanaka, 2008; Kang et al., 2009; Marques-Mari et al., 2009; Yamashiro et al., 2018; Ishikura et al., 2021). Indeed, significant advances during the last 15 years suggest that mouse PSCs can now form the basis for gametogenesis *in vitro*. An important step has been optimization of the ability to generate germ cell-like cells (GCLCs), particularly primordial germ cell-like cells (PGCLCs) and more advanced GCLCs from PSCs (Hayashi et al., 2011; Sasaki et al., 2015; Hwang et al., 2020; Ishikura et al., 2021). Standard protocols for production of PGCLCs from mouse PSCs have been shown to yield GCLCs most similar to endogenous migrating PGCs *in vivo*, which have typically initiated erasure of epigenetic programming inherited from epiblast cells (Seki et al., 2005; Guibert et al., 2012; Seisenberger et al., 2012; Ohta et al., 2017). PGCLCs have been derived from human, monkey, and mouse ESCs and iPSCs carrying female (XX) or male (XY) karyotypes (Hayashi et al., 2011; Sasaki et al., 2015; Sosa et al., 2018; Sakai et al., 2020; Ishikura et al., 2021; Seita et al., 2023).

Although ESCs, which are derived from the inner cell mass (ICM) of the preimplantation embryo, have the advantage of being naturally pluripotent, their use as a treatment for infertility via *in vitro* gametogenesis is not ideal because of ethical issues associated with the requisite destruction of embryos to recover ESCs (Evans and Kaufman, 1981; Nagy et al., 1990), and the fact that resulting offspring will not be biological descendants of the parents since it is not possible to obtain ESCs from adults (Swijnenburg et al., 2005; Guo et al., 2015; Trounson and DeWitt, 2016). In this respect, the induction of germ cell differentiation from iPSCs, which can be derived from adult somatic cells in a patient-specific manner without the need to utilize or destroy embryos, represents a preferable strategy.

iPSCs resemble ESCs but are derived from cells that are not naturally pluripotent. Typically, iPSCs are produced by reprogramming differentiated somatic cells to revert to a pluripotent state. This requires removal of somatic programming from the epigenome. However, somatic epigenomes normally include a stabilized chromatin landscape characterized by highly ordered heterochromatic compartments associated with repressive DNA methylation and histone modifications that preclude expression of genes involved in differentiation pathways other than that which has developed in the particular somatic cell type being used as a source of iPSCs, as well as ordered euchromatic compartments with active modifications that promote expression of genes associated with the unique differentiated fate of the source somatic cell type (Meshorer and Misteli, 2006; Hawkins et al., 2010; Fussner et al., 2011; Atlasi and Stunnenberg, 2017).

Many reports have documented derivation of iPSCs that meet all commonly accepted criteria for pluripotency, but continue to retain “epigenetic memory” manifest as lingering epigenetic programming and associated expression of certain genes normally unique to the source cell type (Chin et al., 2009; Polo et al., 2010; Phetfong et al., 2016). This, in turn, can potentially impact the ability of iPSCs to then be induced to efficiently and/or fully differentiate into a different terminal cell type (Polo et al., 2010; Phetfong et al., 2016). For the therapeutic use of iPSC-derived somatic cell types, less than pristine reprogramming, which may result in a less than optimal but nevertheless sufficiently functional transcriptome, may provide an acceptable means to mitigate certain deleterious somatic conditions in patients, justifying their use in the clinic. However, for purposes of *in vitro* gametogenesis with the objective of generating gametes to be used via assisted reproductive technologies to create offspring, the requirement to recapitulate pristine epigenetic programming and accompanying gene expression associated with normal gametogenesis *in vivo* is obviously much greater.

Cell-type specific gene expression which dictates cell fate and function is regulated by transcription factor (TF) networks functioning within 2-D chromatin landscapes organized into 3-D interactomes (Stadhouders et al., 2018; Stadhouders et al., 2019; Zhong et al., 2023). The 2-D chromatin landscape is manifest from a combination of multiple epigenetic parameters including DNA methylation, histone modifications and non-coding RNAs, all dictating chromatin accessibility (Bannister and Kouzarides, 2011; Guo et al., 2017; Razin and Gavrilov, 2021; Statello et al., 2021). The 3-D interactome is established on the basis of spatial interactions between distant regions of chromosomes (e.g., promoter-enhancer loops) (Furlong and Levine, 2018; Kim and Shendure, 2019; Stadhouders et al., 2019; Hsieh et al., 2020), and between the genome and the nuclear lamina (e.g., topologically associated domains [TADs]) (Guelen et al., 2008; Peric-Hupkes et al., 2010; Kim and Shendure, 2019; Stadhouders et al., 2019). Pristine, cell-type specific epigenetic programming and associated gene expression require the correct integration of all of these parameters.

iPSCs can be derived from differentiated somatic or germ cell types (Takahashi and Yamanaka, 2006; Yamanaka, 2007; Bermejo-Alvarez et al., 2015). We reasoned that if germline cells generated *in vitro* from iPSCs derived from differentiated somatic cell types are impacted by lingering epigenetic memory originating from the source somatic cell type, the same problem should not accrue in germline cells generated from iPSCs initially derived from germline cells, since any lingering epigenetic or transcriptional memory from the source cell type should be directly compatible with the final cell type in the latter scenario. To test this hypothesis, we generated iPSCs from both somatic and germline cells recovered from mice at two different stages—mouse embryonic fibroblasts (MEFs) and PGCs/M-prospermatogonia from male fetuses at embryonic day 13.5 (E13.5), and tail tip fibroblasts (TTFs) and spermatogonial stem cells (SSCs) from male pups at postnatal day 6 (P6). As an initial assessment of the extent to which epigenetic and/or transcriptomic memory was retained in iPSCs derived from somatic versus germ cell types, and subsequently in PGCLCs derived from each set of iPSCs, we examined genome-wide patterns of DNA methylation and gene expression in each resulting cell population. We found that epigenetic and transcriptomic memory reflecting each source cell type were indeed evident in the corresponding iPSCs, but that to a large extent, these effects were no longer detectable in populations of PGCLCs. Ultimately, when compared to endogenous PGCs/M-prospermatogonia at E13.5, the male PGCLCs generated from somatic-cell sourced iPSCs showed no greater differences than did the PGCLCs generated from germline-cell sourced iPSCs, indicating that *in vitro* gametogenesis derived from somatic-cell sourced iPSCs remains a promising approach for the treatment of male infertility.

## Materials and methods

1. Transgenic mouse line—This study required the use of a double transgenic mouse line. We crossed existing lines of transgenic mice to produce males carrying two transgenes: 1) *Id4-eGfp* (*exon1 of the* inhibitor of DNA binding 4 gene ligated to the eGFP marker gene) which drives expression of eGFP in SSCs, and 2) the doxycycline (Dox)-inducible cassette, “*4F2A*,” which encodes the four “Yamanaka” reprogramming factors generated by Carey et al. (2010) in the Jaenisch lab. *Id4-eGfp* transgenic mice were provided by Dr. Jon Oatley (Chan et al., 2014) at Washington State University via Dr. Brian Hermann who had already established a colony of these mice at UTSA. All mice were housed in an AAALAC-approved animal facility under a 12-h light/12-h dark cycle and were provided standard mouse chow and water *ad libitum.* In addition, all experimental procedures involving live mice were preapproved by the UTSA Institutional Animal and Care Use Committee.
2. Recovery of endogenous somatic and germ cell types from double transgenic mice—Briefly, endogenous MEFs were recovered from fetuses at embryonic day (E) 13.5 and enzymatically dissociated as described (Durkin et al., 2013). As described by Khan and Gasser (2016) and Chen et al. (2016), endogenous TTFs were recovered from the tail tips of male mice and plated in MEF medium [DMEM containing 10% heat-inactivated fetal bovine serum, 1% non-essential amino acids (NEAA), 1% GlutaMAX, and 1% Pen Strep] (Gibco by Life Technologies), so that the cells could migrate out from the bone for 7–12 days before being recovered as TTFs. Endogenous M-prospermatogonia (also referred to as E13.5 PGCs) were recovered by fluorescence activated cell sorting (FACS) using SSEA1 (stage-specific mouse embryonic antigen, also known as CD15) and Integrin-β3 (also known as CD61) antibodies to sort for double positive cells as described (Hayashi et al., 2011; Hayashi and Saitou, 2013a, 2013b). Endogenous SSCs were recovered by dissection and enzymatic dissociation of testis tissue from mice at postnatal day 6 (P6), followed by FACS sorting for ID4-eGFP^Bright^ cells as described (Helsel et al., 2017). FACS-sorted ID4-EGFP^Bright^ cells were shown by Helsel et al. (2017) to represent an essentially pure population of SSCs on the basis of validation by the spermatogonial transplantation assay with limiting dilution methodology.
3. Generation and maintenance of mouse iPSCs—Each of the four different endogenous germ and somatic cell types (PGCs/M-prospermatogonia, SSCs, MEFs, TTFs) were placed in culture medium to which DOX was added to induce expression of the reprogramming transgene cassette encoding OCT4, SOX2, KLF4 and C-MYC which, in turn, induced iPS reprogramming as described (Carey et al., 2010; Chen et al., 2016). The medium was then changed to ES medium [DMEM containing 15% embryonic stem cell fetal bovine serum, 1% NEAA, 1% GlutaMAX, 1% Pen Strep, 1,000 U/mL LIF (ESGRO, Merck Millipore), and 0.1 mM β-mercaptoethanol (Gibco by Life Technologies)] on the second day and then cultured for 16–21 additional days and examined for the appearance of ESC/iPSC-like colonies. The medium was then changed every 2 days. When visible colonies appeared, they were transferred to one well each of a 24-well plate for further subculturing in ES medium on CF1 feeder cells (Gibco, A34181). Once each mouse iPSC line was tested to meet the standard criteria of pluripotency, it was then maintained in N2B27 medium with 1,000 U/mL Leukemia Inhibitory Factor (LIF) (Millipore, ESG1107) and 2i (2i = 0.4 μM PD0325901 [MEK/ERK inhibitor] [Stemgent, #04-0006] and 3 μM CHIR99021 [GSK-3 inhibitor] [Biovision, #1677-5] which, together, block the MAPK/Erk pathway and glycogen synthase kinase 3 to inhibit mouse ESCs from responding to differentiation-inducing signals), in wells coated with 0.1% (w/v) gelatin (Millipore, #ES-006-B). Cells were split and placed in fresh medium every 3 days. N2B27 medium is composed of DMEM/F12 + N2 and Neurobasal + B27. For DMEM/F12+N2, 495 mL DMEM/F12 (Gibco, #21041-025) were mixed with 5 mL of N2. N2 is composed of 0.5 mL of 25 mg/mL insulin stock solution (Sigma-Aldrich, #I-1882), 0.5 mL of 100 mg/mL apo-transferrin stock solution (Sigma-Aldrich, #T-1447), 0.33 mL of 7.5% BSA solution (Gibco, #15260-037), 16.5 μL of 0.6 mg/mL of progesterone stock solution (Sigma-Aldrich, #P8783), 50 μL of 160 mg/mL putrescine stock solution (Sigma-Aldrich, #P5780), and 5 μL of 3 mM sodium selenite stock solution (Sigma-Aldrich, #S5261). For Neurobasal+B27, 480 mL Neurobasal were mixed with 10 mL of B27 (Gibco, #12587-010), 5 mL of penicillin-streptomycin (Gibco, #15070-063), and 5 mL of Glutamax (Gibco, #35050-061). For 1 L of N2B27 medium, 500 mL DMEM/F12 + N2 were mixed with 500 mL Neurobasal + B27, and then 1.8 mL of β-mercaptoethanol (Gibco, #21985-023) were added. For stock preparation, 40 mL aliquots of N2B27 were stored at −80°C. After adding 2i and LIF in N2B27, medium could be stored at 4°C for up to 2 weeks.
4. Characterization/validation of mouse iPSCs—To validate each iPSC line, characterization of karyotypes was outsourced to Cell Line Genetics Inc. and WiCell Laboratory. Expression of pluripotency markers was assessed in house by qRT-PCR and immunocytochemistry (ICC) to detect pluripotency factor expression at the RNA and protein levels, respectively. Antibodies for OCT4 (abcam, #ab19857), SOX2 (abcam, #ab97959), NANOG (abcam, #ab80892), SSEA1 (abcam, #ab16285), as well as Alkaline Phosphatase staining (Vector Laboratories, Inc., #SK-5100) were used to detect pluripotency markers at the protein level. qRT-PCR was used to detect expression of *Pou5f1*, *Sox2*, *Nanog*, and *Klf4* at the RNA level in association with detection of housekeeping gene transcripts—*Gapdh*—which was used to calculate ΔCt values. qRT-PCR primer sequences are shown in Supplementary Table S1.
5. Induction of mouse PGCLCs *in vitro*—The protocol for PGCLC differentiation was adapted from Dr. Mitinori Saitou’s laboratory as described (Hayashi et al., 2011; Hayashi and Saitou, 2013b). Briefly, mouse epiblast-like cells (EpiLCs) were induced from 1.0 × 10^5^ mouse iPSCs in wells of a 12-well plate coated with 16.7 μg/mL human plasma fibronectin (Life Technologies, #13256-029) in EpiLC medium [(N2B27 containing 20 ng/mL activin A (PeproTech, #120-14), 12 ng/mL bFGF (Gibco, #13256-029), and 1% Knockout Serum Replacement (KSR) (Gibco, #10828-028)]. The EpiLC medium was changed the next day after plating. On day 2, EpiLCs were collected and a total of 2.0 × 10^5^ EpiLCs were plated in each well (2000 cells per well) of a low-cell-binding U-bottom 96-well Nunclon Sphera Microplate (Thermo Fisher Scientific, #174925). Then, mouse PGCLCs were induced from the EpiLCs described above in the 96-well microplate under floating conditions in mouse PGCLC medium also referred to as GK15 [Glasgow’s Minimal Essential Medium (GMEM) (Gibco, #11710-035) with 15% KSR, 0.1 mM NEAA (Gibco, #11140-050), 1 mM sodium pyruvate (Gibco, #11360-070), 0.1 mM 2-mercaptoethanol (Gibco, #21985-023), 1% penicillin-streptomycin (Gibco, #15070-063), and 2 mM GlutaMAX (Gibco, #35050-061)], plus 500 ng/mL BMP4 (R&D Systems, #314-BP-500), 1,000 U/mL LIF, 100 ng/mL SCF (R&D Systems, #455-MC-500), and 50 ng/mL EGF (R&D Systems, #2028-EG-200) for 4 days without changing the medium. Putative PGCLC aggregates were then collected on day 4 for FACS.
6. FACS to recover enriched populations of mouse PGCLCs—Day 4 floating aggregates were incubated in 0.05% Trypsin-EDTA (Gibco, #25300-054) for 6–8 min at 37°C with periodic pipetting and the trypsin was then quenched with a 5X volume of DMEM medium (Gibco, #11960-069) containing 10% FBS followed by pipetting to generate a single cell suspension. Then the cell suspension was filtered through a 70 µM nylon cell strainer (FALCON, #352350) and centrifuged at 1,000 rpm for 5 min. After centrifugation, the supernatant was discarded. The cell pellet was re-suspended in DPBS (Gibco, #1130-082) containing 10% FBS and counted for viable cells. Next, the viable cells were incubated with 0.25 µg of TruStain FcX™ PLUS (anti-mouse CD16/32) (BioLegend, #156603) antibody per 10^6^ cells in 100 µL for 5–10 min on ice to block Fc receptors. The cells were then centrifuged at a speed of 1,000 rpm at 4°C for 5 min and the supernatant was removed. The cell pellet was resuspended at a density of 10^6^ cells per 100 µL DPBS with 10% FBS, 1 µL of PE anti-mouse CD61 (Integrin β3) antibody (BioLegend, #104307), and 5 µL of Brilliant Violet 421 anti-mouse CD15 (SSEA-1) fluorescent antibody (BioLegend, #125613) and incubated on ice for 20 min in the dark. After incubation, cells were washed twice with 1 mL of DPBS with 10% FBS and centrifuged at 1,000 rpm for 5 min. Cell pellets were resuspended in an appropriate volume of DBPS with 10% FBS and 5 µL of propidium iodide (PI) (BioLegend, #421301) was added per 10^6^ cells to create a viable staining solution that allowed us to exclude dead cells during sorting. A CD61+/CD15+ double positive population was sorted by FACS (BD Biosciences, FACS Aria II) and collected in a solution of DPBS plus 10% FBS.
7. Characterization/validation of enriched PGCLC populations—To validate each PGCLC population, expression of pluripotency and germ cell markers was assessed in-house by qRT-PCR and ICC to detect pluripotency and germ cell factor expression at the RNA and protein levels, respectively. Antibodies for OCT4 (abcam, #ab19857), NANOG (abcam, #ab80892), AP-2γ (Santa Cruz, #SC-12762), and PRDM1 (Cell Signaling Technology, # 9115S) were used to detect pluripotency and germ cell markers at the protein level. qRT-PCR was used to detect expression of *Pou5f1*, *Sox2*, *Nanog*, *Fgf5*, *Dnmt3b*, *Wnt3*, *Dazl*, *Dnd1*, *Dppa3*, *Itgb3*, *Nanos3*, *Prdm1*, *Prdm14*, and *Tfap2c* at the RNA level in association with detection of a housekeeping gene transcript—*Gusb*—which was used to calculate ΔCt values. qRT-PCR primer sequences are shown in Supplementary Table S2.
8. Genomic DNA extraction—To assess the status of DNA methylation and gene expression in a coordinated manner in each cell type, DNA and RNA were isolated in parallel from each cell sample. Thus, a portion of cells from each sample was used for RNA isolation and the other portion was used to extract genomic DNA. The iPSCs were cultured in ES medium with 15% FBS on feeder cells for 10 passages and then transferred to N2B27 media with 2i + LIF in feeder free conditions for 4 passages before being collected for DNA and RNA extraction. Cells were lysed in cell lysis buffer with 10 µL of 20 mg/mL Proteinase K solution (final volume 200 µL) and then incubated for 4 h to overnight at 55°C inverting occasionally to mix. Digested cells were transferred to phase lock microcentrifuge tubes and then 200 µL of phenol:chloroform was added and the tubes were shaken rapidly to form an emulsion. The tubes were then centrifuged at 13,000xg at 21–25°C for 5 min. Then 200 µL of the aqueous phase was transferred into a fresh microcentrifuge tube and 20 µL of sodium acetate together with 1 µL of linear polyacrylamide (LPA) (Sigma-Aldrich, #56575) was added. Cold 100% ethanol in a 1:2–1:3 ratio (400–600 µL) was then added and the tubes were placed at −20°C overnight. On the second day, the solution was centrifuged at maximum speed (at least 13,000xg) for 30 min at 4°C. The supernatant was then removed without disturbing the slippery pellet of DNA at the bottom of the tube. Then, 600 µL of cold 75% ethanol was added and mixed well to wash the DNA by rapidly shaking the tube. The tube was then centrifuged again at maximum speed (at least 13,000xg) for 30 min. This wash and centrifuge process was then repeated 2–3x. Following the final wash, the supernatant was removed, and the pellet was air-dried for 5–15 min. After the DNA pellet was completely dried, it was resuspended in 50 µL of TE buffer and incubated at 55°C for 30–60 min to ensure the DNA was completely dissolved. Genomic DNA was then purified using Genomic DNA Clean & Concentrator-10 (Zymo Research, #D4011) according to the manufacturer’s instructions.
9. RNA-seq libraries and analysis—In preparation for bulk RNA-seq, cells were lysed in TRI reagent and total RNA was purified using a Direct-zol RNA Miniprep kit (Zymo Research, #R2050) according to the manufacturer’s instructions. Total RNA was then incubated with 2 μL of RQ1 DNase (supplied at a concentration of 1 U/μL, Promega, #M6101) per 1 μg RNA for 30 min at 37°C and was then re-purified using RNA Clean & Concentrator-5 (Zymo Research, #R1016) according to the manufacturer’s instructions. About 50 ng of total RNA from each sample was used for synthesis and amplification of cDNA. cDNA synthesis was performed by the UTSA Genomics Core using the QuantSeq 3′ mRNA-Seq Library Prep Kit FWD from Illumina (LEXOGEN). The resulting cDNA libraries were outsourced to the North Texas Genome Center (Arlington, Texas) for sequencing on an Illumina NovaSeq 6000 platform. All reads from the QuantSeq sequencing datasets were processed with cutadapt v1.18 to remove adaptors and poly-A sequences. Reads of 30-bp or longer were mapped to the GRCm39 version of the mouse reference genome using Rsubread v2.10.1. RNA-seq data from SSCs was downloaded from the NCBI database, SRA ftp site (Cheng et al., 2020), and mapped onto the mouse genome GRCm39 using Rsubread v2.10.1. The QuantSeq and SSC RNA-seq datasets were processed using the R software (version 4.1.1) packages edgeR v3.38.1 and DESeq2 v1.36 with default settings to detect and analyze differential gene expression levels, including <5% for false discovery rate and *p* < 0.05. Data from biological replicates was averaged and differentially expressed genes were defined as those with a ≥1.5-fold difference in expression levels between the control ESC line and each set of iPSC lines produced in this study. Hierarchical clustering was performed using the hclust function (Murtagh and Legendre, 2014) and principal component analysis (PCA) was performed using the autoplot and prcomp function without scaling (R Core Team, 2019).
10. Whole-Genome Bisulfite Sequencing (WGBS) libraries and analysis—About 50 ng of genomic DNA from each sample was subjected to bisulfite conversion. Preparation of the WGBS libraries was done using the Pico Methyl-Seq Library Prep Kit (Zymo Research, #D5456) per the manufacturer’s instructions. The libraries were then outsourced to Novogene Corporation Inc. (Sacramento, California) and the North Texas Genome Center (Arlington, Texas) for sequencing on an Illumina HiSeq and NovaSeq 6000 platform, respectively. All reads from WGBS data were processed with Trim Galore v0.6.5 to remove adaptors and the first four bases. Reads were mapped onto the mouse reference genome GRCm39 using bwa-meth and PCR duplicates were removed with the Picard tool. The analysis of differentially methylated regions (DMRs) was performed using wg-blimp workflow as previously described (Woste et al., 2020). Per the criteria established by Hansen et al. (2012) and Jühling et al. (2016), a DMR in this study was defined as a region, variable in length, containing at least 5 CpG sites displaying a ≥30% difference in the level of DNA methylation value when compared to a reference control. In this study the reference control was a validated pluripotent ESC line. Methylation over a region was calculated for each CpG in the region and then these individual values were averaged to give a representative value for the region. Hierarchical clustering was performed using the clusterSamples function and PCA was performed using the PCASamples function from methylKit v1.22.0 using the percent methylation matrix as an input (Akalin et al., 2012).

## Results

**FIGURE 1:**
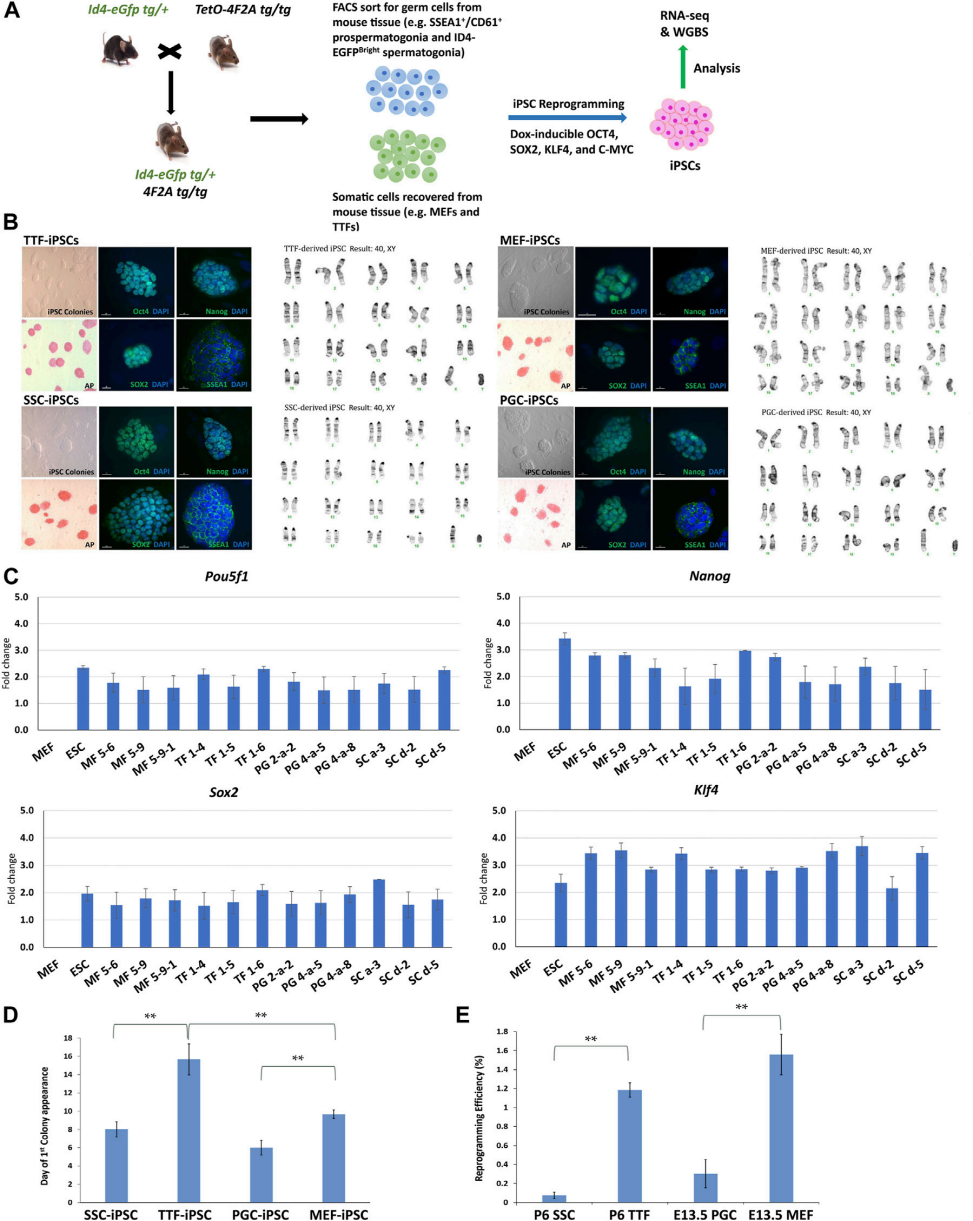
Characterization of TTF-, SSC-, MEF-, and PGC/M-prospermatogonia-derived iPSC lines. **(A)** Experimental scheme of iPSC derivation from different somatic and germ cell types. Somatic cell types and endogenous germ cell types were isolated from double-transgenic mice at either embryonic day 13.5 (E13.5) or postnatal day 6 (P6) and induced to form iPSCs by DOX-driven expression of the *4F2A* reprogramming cassette transgene. **(B)** Immunocytochemistry (ICC) and karyotype analysis of TTF-, MEF-, SSC-, and PGC/M-prospermatogonia-derived iPSC lines. Normal karyotypes, colony morphology, alkaline phosphatase (AP) staining, and ICC staining for mouse pluripotency markers: OCT4, SOX2, NANOG, and SSEA1 for each iPSC line are shown. **(C)** Quantification of pluripotency gene expression at the RNA level in each iPSC line. Gene expression profiles for each iPSC line were measured by qRT-PCR. For each gene, the ΔCT from the CT value of the control housekeeping gene, *Gapdh* was calculated. Then, the ΔΔCT from the CT value from the negative control MEFs was calculated and set at zero. Fold change is shown on the Y-axes in log_2_ scale. **(D,E)** Bar graphs illustrate the timing of first colony appearance in iPSC lines derived from each source cell type, and the efficiency of reprogramming for each starting cell type. Data are represented as means ± SEMs (*n* = 3). *p < 0.05, **p < 0.01, one-way ANOVA, followed by Tukey’s test.

Generation and characterization of iPSC lines derived from differentiated somatic and germ cell types. In order to most conveniently generate iPSC lines, we crossed existing lines of transgenic mice to produce males carrying two transgenes: an SSC marker transgene, *Id4-eGFP* (Chan et al., 2014), and a polycistronic cassette transgene, *4F2A*, encoding the four “Yamanaka factors” required to induce reprogramming to a pluripotent state regulated by a doxycycline (DOX)-inducible promoter (Carey et al., 2010) (Figure 1A). From this double transgenic line of mice, we derived iPSCs made from two different somatic and two different germ cell sources—somatic MEFs and germline PGCs/M-prospermatogonia—both recovered from male fetuses at E13.5, and somatic TTFs and germline SSCs—both recovered from male pups at P6 (Figure 1A). PGCs/M-prospermatogonia and SSCs were recovered by FACS sorting dissociated testis cells at E13.5 or P6, respectively. Recovery of PGCs/M-prospermatogonia at E13.5 was based on sorting integrin-β3 (CD61) and SSEA1 (CD15) double-positive cells, while recovery of SSCs at P6 was based on sorting ID4-eGFP^Bright^ cells, both as described (Durcova-Hills et al., 1999; Hayashi et al., 2011; Oatley et al., 2011; Chan et al., 2014). MEFs were recovered from E13.5 male fetuses and enzymatically dissociated as described (Durkin et al., 2013). TTFs were recovered from the tail tips of P6 male pups and plated in MEF medium allowing fibroblasts to migrate out from the tail tissue for 7–12 days before being recovered as TTFs (Chen et al., 2016; Khan and Gasser, 2016).

Three separate iPSC lines were generated from each source cell type: MF56, MF59, and MF591 from MEFs, TF14, TF15, and TF16 from TTFs, PG2a2, PG4a5, and PG4a8 from PGCs/M-prospermatogonia, and SCa3, SCd2, and SCd5 from SSCs. All iPSC lines expressed mouse pluripotency markers at the protein level, including alkaline phosphatase (AP), OCT4, SOX2, NANOG and SSEA1, and all showed normal karyotypes and standard mouse iPSC colony morphology, including compact dome-shaped refractile colonies (Takahashi and Yamanaka, 2006) (Figure 1B). Additional validation of pluripotency of these iPSC lines was based on expression of pluripotency markers at the RNA level detected by qRT-PCR (Figure 1C). This data demonstrates that the iPSC lines derived from all four source cell types met all standard criteria of pluripotency.

Reprogramming efficiency—defined as the number of AP+ colonies divided by the starting density of 8,000 cells at the time of DOX induction of reprogramming converted to a percentage—was calculated for each line. The reprogramming efficiency and timing of first iPSC colony appearance differed for lines derived from each source cell type. Thus, the timing of first iPSC colony appearance was approximately 6 days for PGC/M-prospermatogonia-derived iPSCs, 8 days for SSC-derived iPSCs, 10 days for MEF-derived iPSCs, and 16 days for TTF-derived iPSCs after DOX induction in each case (Figure 1D). As expected, iPSCs derived from germ cell types required less time for initial iPSC colony appearance compared to iPSCs derived from somatic cell types. However, somewhat surprisingly, the somatic cell types reprogrammed to pluripotency more efficiently than the germ cell types (from highest to lowest): MEFs > TTFs > PGCs/M-prospermatogonia > SSCs (Figure 1E). Importantly, this may simply represent differences in the proliferative status of each starting cell type and/or culture conditions which initially involved media optimized for each source cell type before transitioning each into iPSC reprogramming media. In addition, PGCs/M-prospermatogonia at E13.5 are normally poised to enter a stage of cell cycle arrest, so may therefore be initially refractory to proliferative signals in the iPSC reprogramming media.

**FIGURE 2:**
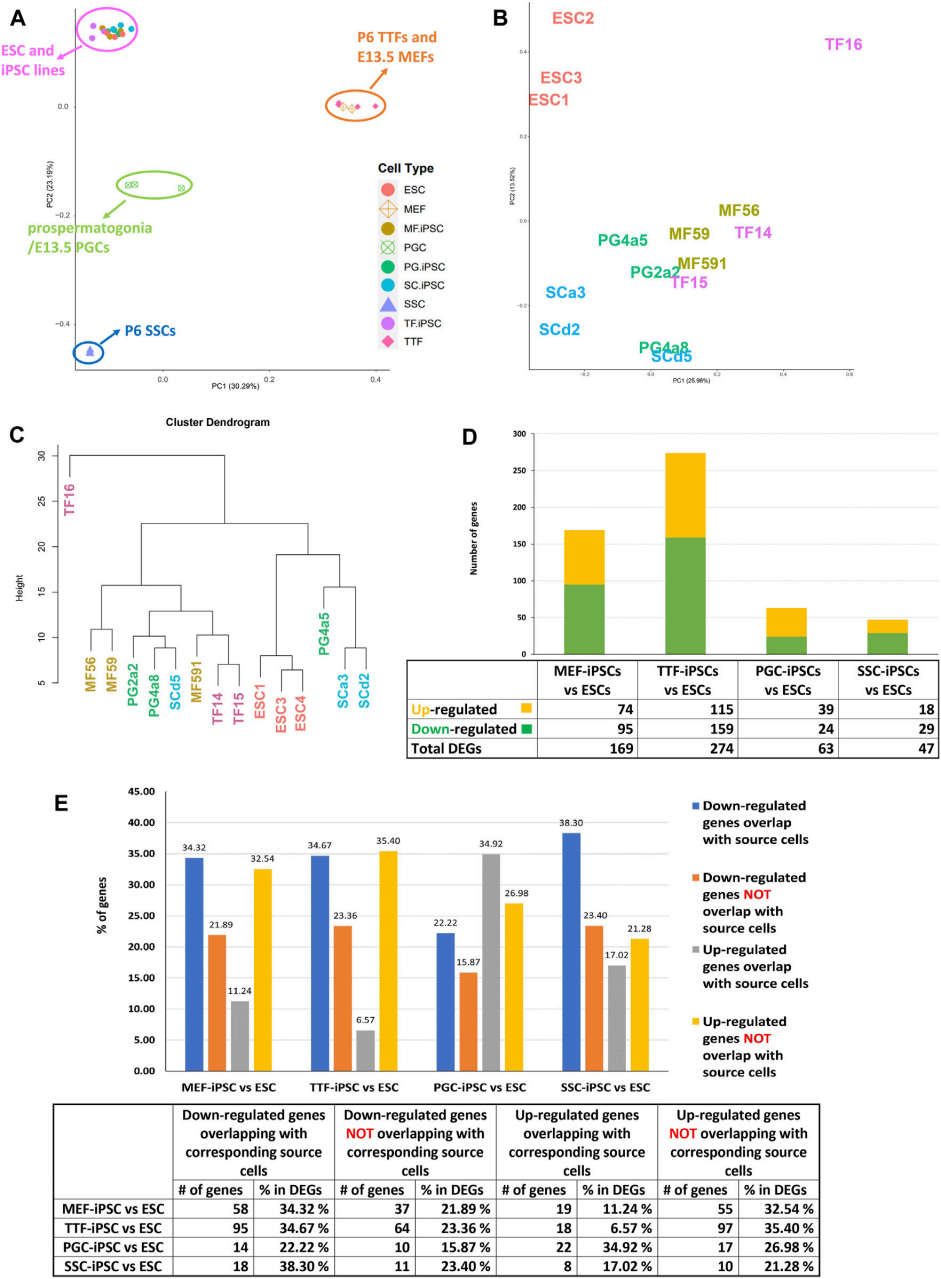
Global analysis of RNA-seq transcriptome profiles of iPSC lines and corresponding source cell types. **(A)** A PCA plot of transcription profiles in endogenous MEFs, TTFs, PGCs/M-prospermatogonia, and SSCs recovered/derived directly from living mice, along with three replicates each of the four types of iPSC lines and three replicates of the control ESC line. **(B)** A second PCA analysis was focused strictly on the 12 iPSC and 3 ESC line replicate transcription profiles. **(C)** A hierarchical clustering of the 15 PSC transcription profiles was generated based on 1-Pearson’s correlation distance. **(D)** Quantification of bulk RNA-seq data revealed differentially expressed genes (DEGs) in each iPSC group compared to the control mouse ESC line. **(E)** A bar graph shows the percentage of upregulated and downregulated DEGs from the RNA-seq data for each iPSC group, and the extent to which that correlates with DEGs in the corresponding source cell type, when each was compared to the control ESC line. Differential gene expression = ≥1.5x fold change, FDR < 5%, *p* < 0.05, and *n* = 3 in each group.

iPSCs retain transcriptional memory from their source cell types. We used RNA-seq to compare the transcriptomes of the iPSC lines established from each differentiated somatic or germline source cell type with triplicate isolates of a validated pluripotent ESC line (V6.5, Novus Biologicals, #NBP1-41162) (Figure 2). We also analyzed each corresponding source cell type from which each set of iPSC lines were derived. A two-dimensional principal component analysis (PCA) shows that while transcriptomes of the iPSC lines derived from all four source cell types were much more similar to that of the control pluripotent ESC line than to those of the source differentiated cell types (Figure 2A), the iPSC transcriptomes were not completely identical to the ESC transcriptome (Figures 2B–D and Supplementary Figure S1). Thus, our control naïve ESC line displayed consistent differences in gene expression patterns relative to iPSC lines derived from any of the four source cell types we tested. Further, distinctions could be detected among the iPSC lines which appeared to be more related to the source cell type than to the developmental stage/age of the source cells. Importantly, the extent of variation observed between each group of iPSCs and the control ESC line was consistently greater than that observed among the three replicates of the control ESC line. Thus, there were small but consistent differences in gene expression patterns that distinguished the iPSC lines from the control ESC line.

To determine if the differences in transcriptome patterns we observed between our iPSC lines and the control ESC line reflected transcriptional memory from the source cell types of each iPSC line, we compared DEGs between the control ESC line and 1) each set of triplicate iPSC lines, and 2) each set of triplicate samples from the corresponding differentiated source cell types (Table 1). Among the differentiated source cell types, the less differentiated germline PGCs/M-prospermatogonia transcriptome showed the greatest similarity to the control ESC line, while the more differentiated germline SSC transcriptome showed the least similarity to the control ESC line. Among the two differentiated somatic cell types, the less differentiated MEF transcriptome was more similar to the control ESC transcriptome than the more differentiated TTF transcriptome. Thus, as expected, less differentiated cell types more closely resembled the pluripotent ESC control than more differentiated cell types.

**TABLE 1:** DEGs in source cells and corresponding iPSCs^a^.

# of DEGs groups and types of DEGs		Source cells vs. ESCs	iPSCs vs. ESCs	DEGs that were common to both
MEFs/MEF-iPSCs	Upregulated	2,951	74	19
	Downregulated	3,337	95	58
TTFs/TTF-iPSCs	Upregulated	3,517	115	18
	Downregulated	3,906	159	95
PGCs/PGC-iPSCs	Upregulated	2,615	39	22
	Downregulated	2,640	24	14
SSCs/SSC-iPSCs	Upregulated	4,691	18	8
	Downregulated	4,061	29	18

^a^ Differential gene expression = ≥1.5x fold change, FDR < 5%, *p* < 0.05, and *n* = 3 in each group.

In each case, a substantial subset of the DEGs distinguishing each set of iPSC lines from the ESC control line was also found among DEGs distinguishing the corresponding source cell type from the control ESC line (Figure 2E; Table 1; Supplementary Table S3). Specifically, 46%, 41%, 57% and 55% of DEGs distinguishing the MEF-, TTF-, PGC- and SSC-derived iPSC lines, respectively, from the control ESC line were also among the DEGs distinguishing the corresponding source cell types from the control ESC line, indicative of significant transcriptional memory persisting during derivation of iPSCs from each source cell type (Table 1 and Supplementary Figure S1). Interestingly, there was a higher proportion of retention of source cell-type specific DEGs in iPSC lines derived from germline cell types than in iPSC lines derived from somatic cell types, however the proportion of DEGs distinguishing each set of iPSC lines from the control ESC line was substantial (>40%) in all four sets of iPSCs.

Among the upregulated DEGs found in both iPSCs and their corresponding source cell types, we identified three groups of biologically interesting genes (Supplementary Table S3). The first group included genes that were unique to the MEF and TTF groups, including genes differentially expressed in either MEFs only, TTFs only or both MEFs and TTFs relative to the control ESCs. These appeared to represent somatic/fibroblast lineage-specific genes. The second group included genes that were unique to the PGC/M-prospermatogonia and SSC groups, including genes differentially expressed in either PGCs/M-prospermatogonia only, SSCs only or both PGCs/M-prospermatogonia and SSCs relative to the control ESCs. These appeared to represent germline lineage-specific genes. The third group included genes differentially expressed in both the somatic and germ cell groups relative to the control ESCs, and thus appeared to represent genes indicative of a general differentiated state. In all three cases, the persistent expression of these genes that are normally expressed in one or more of the differentiated cell types used as sources of these sets of iPSCs, but not in true pluripotent cell types such as the control ESCs, appears to represent retention of transcriptional memory during derivation of iPSCs.

**FIGURE 3:**
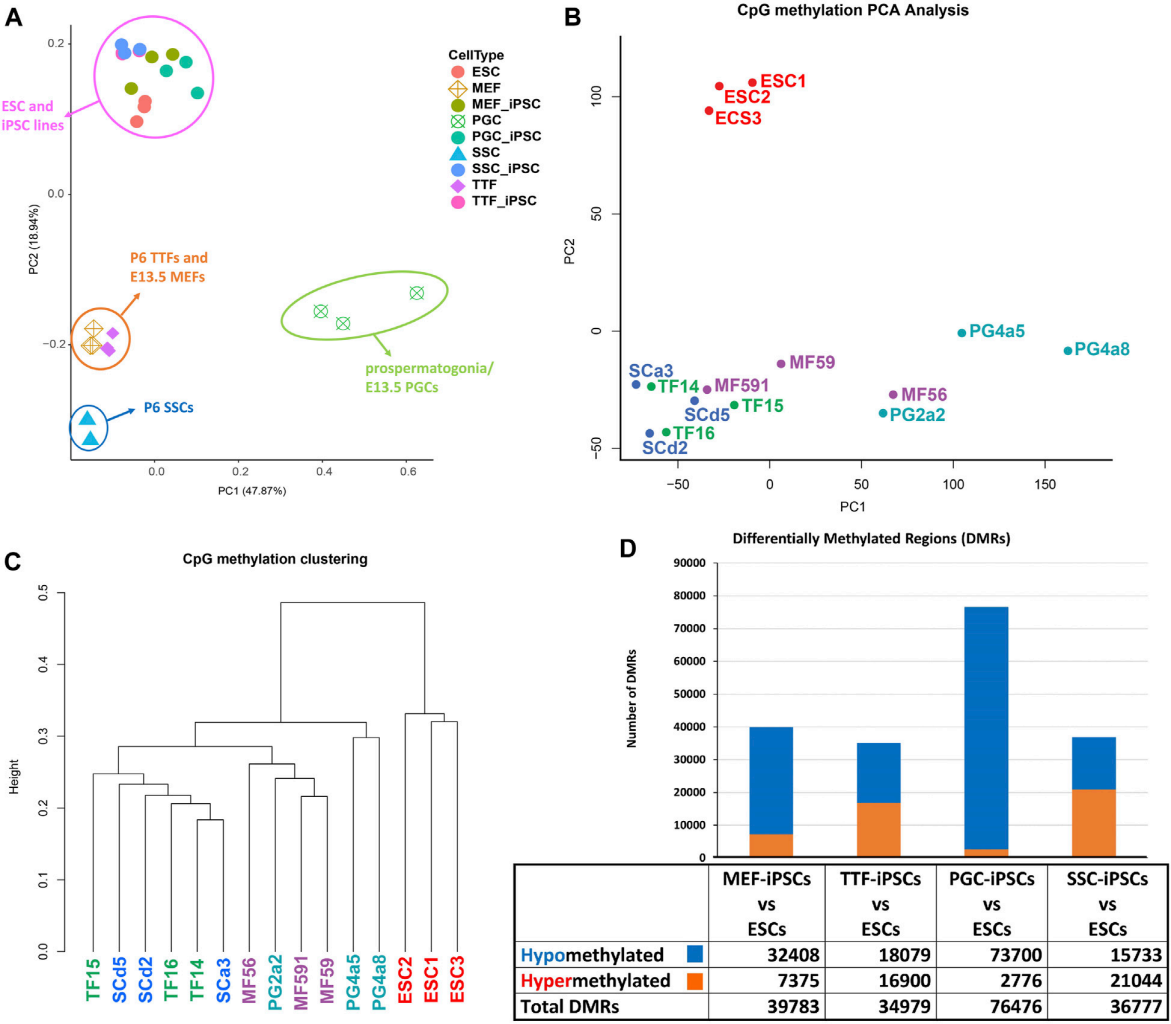
Analysis of global DNA methylation profiles in iPSC lines and corresponding source cell types. **(A)** A PCA of CpG DNA methylation profiles in source differentiated cell types, derived iPSC lines and control ESC lines based on whole-genome bisulfite sequencing (WGBS) data shows that the pluripotent control ESCs and derived iPSCs cluster together, separated from each differentiated source cell type. **(B)** A PCA of CpG DNA methylation profiles in iPSC lines and control ESC lines shows that there are distinctions between the control ESC lines and the derived iPSC lines. **(C)** Hierarchical clustering of CpG DNA methylation profiles in the derived iPSC lines and the control ESC lines is based on 1-Pearson’s correlation distance. **(D)** Quantification of differentially methylated regions (DMRs) in each iPSC group is compared to the control ESC lines. Differential methylation differences of <30% within regions of ≥300 bp of ≤5 CpGs were excluded.

iPSCs retain epigenetic memory from their source cell types. Differential cell-type specific gene expression is often tied to differential cell-type specific epigenetic programming. DNA methylation is perhaps the most extensively studied epigenetic mark. The presence of DNA methylation is commonly found in promoters and enhancers associated with transcriptionally repressed genes, whereas its absence in those same regulatory regions is often associated with actively transcribed genes (Lande-Diner and Cedar, 2005; Berger, 2007; Moore et al., 2013). To assess potential persistence of epigenetic memory in iPSC lines established from each somatic or germline source cell type, we compared the methylome from the control ESC line with those from each differentiated source cell type and each corresponding set of iPSC lines, and identified differentially methylated regions (DMRs) as described (Woste et al., 2020) (Figure 3). PCA analysis of DNA methylation patterns of the different sets of iPSCs relative to the control ESCs and source cell types showed that the iPSC lines clustered with the control ESC line and were clearly distinct from each source cell type (Figure 3A). However, when the four sets of iPSCs were directly compared to the control ESC line, they were distinct, indicating lingering epigenetic differences in the iPSCs relative to the validated pluripotent ESC line (Figure 3B). Thus, among the iPSC DNA methylation datasets, three distinct clades could be discerned (Figure 3C). Interestingly, these distinct clades appeared to be segregated more by stage/age of the source cell types than by identity of the source cell types.

In addition to investigating overall patterns of DNA methylation in each set of iPSC lines by PCA and hierarchical clustering, we also distinguished DMRs detected in each set of iPSCs based on their hypo- or hypermethylated status relative to the control ESCs (Figure 3D). Surprisingly, when compared to the control ESC line, DMR patterns were most similar in the TTF- and SSC-derived iPSCs, less so in the MEF-derived iPSCs, and least similar in the PGC-derived iPSCs based on the total number of DMRs detected in each case. Moreover, the DMRs in the PGC/M-prospermatogonia-derived iPSCs were generally hypomethylated relative to these same sites in the control ESCs, suggesting these iPSC lines reflect the more hypomethylated status of the PGCs/M-prospermatogonia from which they were derived compared to that seen in endogenous inner cell mass cells from which the control ESC line was derived. This reflects the observation that developing germ cells reach what has been termed the “epigenetic ground state” with less genome-wide DNA methylation than any other cell type at any other stage during the lifespan of the individual (Hajkova, 2011). Finally, the SSCs, which are known to have gained significant genome-wide *de novo* DNA methylation during their development from PGCs/M-prospermatogonia (Lee et al., 2014) showed more hypermethylated DMRs than hypomethylated DMRs when compared to the ESCs. Thus, as was the case with the differing patterns of gene expression we observed among the different groups of iPSCs when each was compared to the control ESC line, it appears that the DNA methylation pattern of the starting cell type can predispose a unique DNA methylation profile in subsequently derived iPSCs. This same concept was also supported by our observation that the MEF-derived iPSCs showed more hypomethylated DMRs compared to the TTF-derived iPSCs when these were compared to the ESCs. This appears to reflect the fact that MEFs recovered from E13.5 embryos have more recently undergone resetting of global DNA methylation levels than have TTFs recovered from pups at P6. Together, these represent apparent examples of epigenetic memory persisting from a starting cell type throughout the reprogramming process required to generate iPSCs.

**FIGURE 4:**
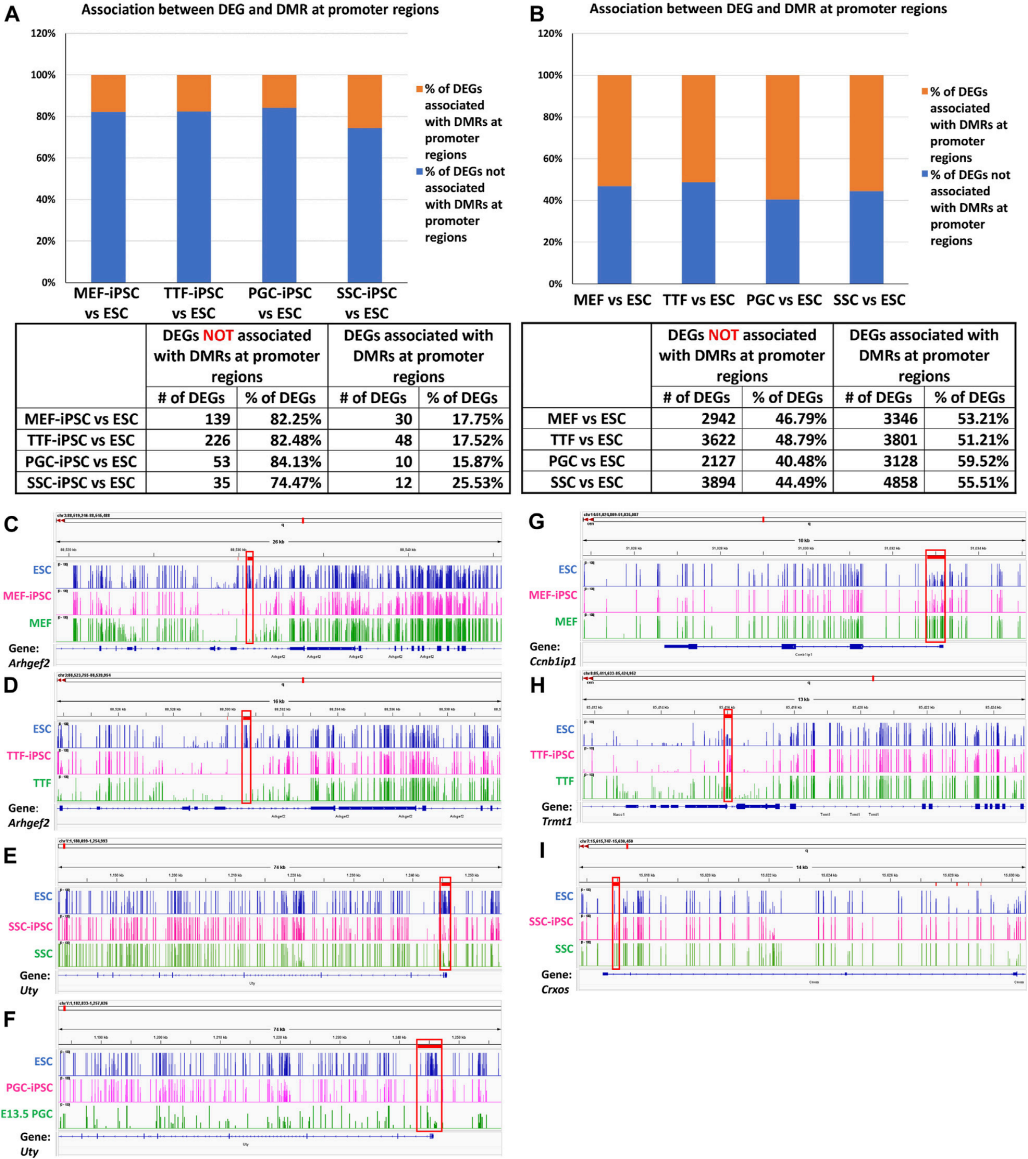
Methylation patterns in promoter regions of genes differentially expressed in source cell types and subsequently derived iPSCs. **(A,B)** Bar graphs display percentages of DEGs that correlate with promoter region DMRs in both source cells (left) and corresponding derived iPSC lines (right), relative to ESCs. (Promoter region = 900-bp upstream to 500-bp downstream of the transcription start site). Integrative Genomics Viewer snapshots of DNA methylome data showing hypomethylated **(C–F)** and hypermethylated **(G–I)** DMRs from **(C)** ESCs, MEF-iPSCs and MEFs near the *Arhgef2* locus; **(D)** ESCs, TTF-iPSCs, and TTFs near the *Arhgef2* locus; **(E)** ESCs, PGC-iPSCs, and PGCs near the *Uty* locus; **(F)** ESCs, SSC-iPSCs, and SSCs near the *Uty* locus; **(G)** ESCs, MEF-iPSCs and MEFs near the *Ccnb1ip1* locus; **(H)** ESCs, TTF-iPSCs, and TTFs near the *Trmt1* locus; and **(I)** ESCs, SSC-iPSCs, and SSCs near the *Crxos* locus. Each track spans percent methylation values from 0% to 100% on the Y-axis and genic regions from −900 bp to +500 bp relative to the transcriptional start site on the X-axis. Tracks are colored by cell types: blue = ESCs, pink = iPSCs, green = starting cell types. Red boxes = DMRs found at promoter regions.

Relationship between differential epigenetic programming and differential gene expression in iPSCs derived from different source cell types. Since DNA methylation plays an important role in regulating transcriptional activity (Moore et al., 2013), we mined our DMR and DEG datasets to identify genes that were both differentially expressed and showed DMRs in their promoter regions when each set of iPSCs was compared to the control ESCs (Figures 4A, B). Interestingly, we observed a higher association between DEGs and promoter region DMRs when the differentiated source cells were compared to the control ESCs (50%–60%), than when the corresponding iPSC lines were compared to the ESCs (12%–26%). This aligns with the suggestion that DNA methylation plays a more important role in stabilizing commitment to lineage-specific differentiation than in reprogramming differentiated cells back to pluripotent cells (Jackson et al., 2004; Ji et al., 2010; Khavari et al., 2010; Maruyama et al., 2011; Bock et al., 2012).

DNA methylation browser tracks are shown for exemplary DEGs in Figures 4C–I. The *Arhgef2* gene for the MEF and TTF groups and the *Uty* gene for the PGCs/M-prospermatogonia and SSC groups were selected from the gene list in Supplementary Table S3 as examples of DEGs upregulated in iPSCs relative to the control ESCs. As shown in Figures 4C–G, the promoter regions of these genes also exhibited lower DNA methylation levels in the iPSCs relative to the control ESCs. Similarly, *Ccnb1ip1* from the MEF-iPSCs, *Trmt1* from the TTF-iPSCs, and *Crxos* from the SSC-iPSCs were genes selected from Supplementary Table S3 as examples of DEGs downregulated in iPSCs relative to the control ESCs. These genes exhibited elevated promoter-region DNA methylation levels in the iPSCs relative to the control ESCs (Figures 4G–I). Together, the genes shown in Figures 4C–I represent examples of DEGs that showed expected differential DNA methylation levels corresponding to differential expression levels, consistent with the expected correlation of hypomethylation of DNA with open chromatin that is permissive of active transcription and hypermethylation of DNA with closed chromatin that represses active transcription (Lande-Diner and Cedar, 2005; Berger, 2007). Thus, these examples are consistent with the concept that epigenetic memory inherited from the source cells can directly contribute to differential gene expression in iPSCs.

**FIGURE 5:**
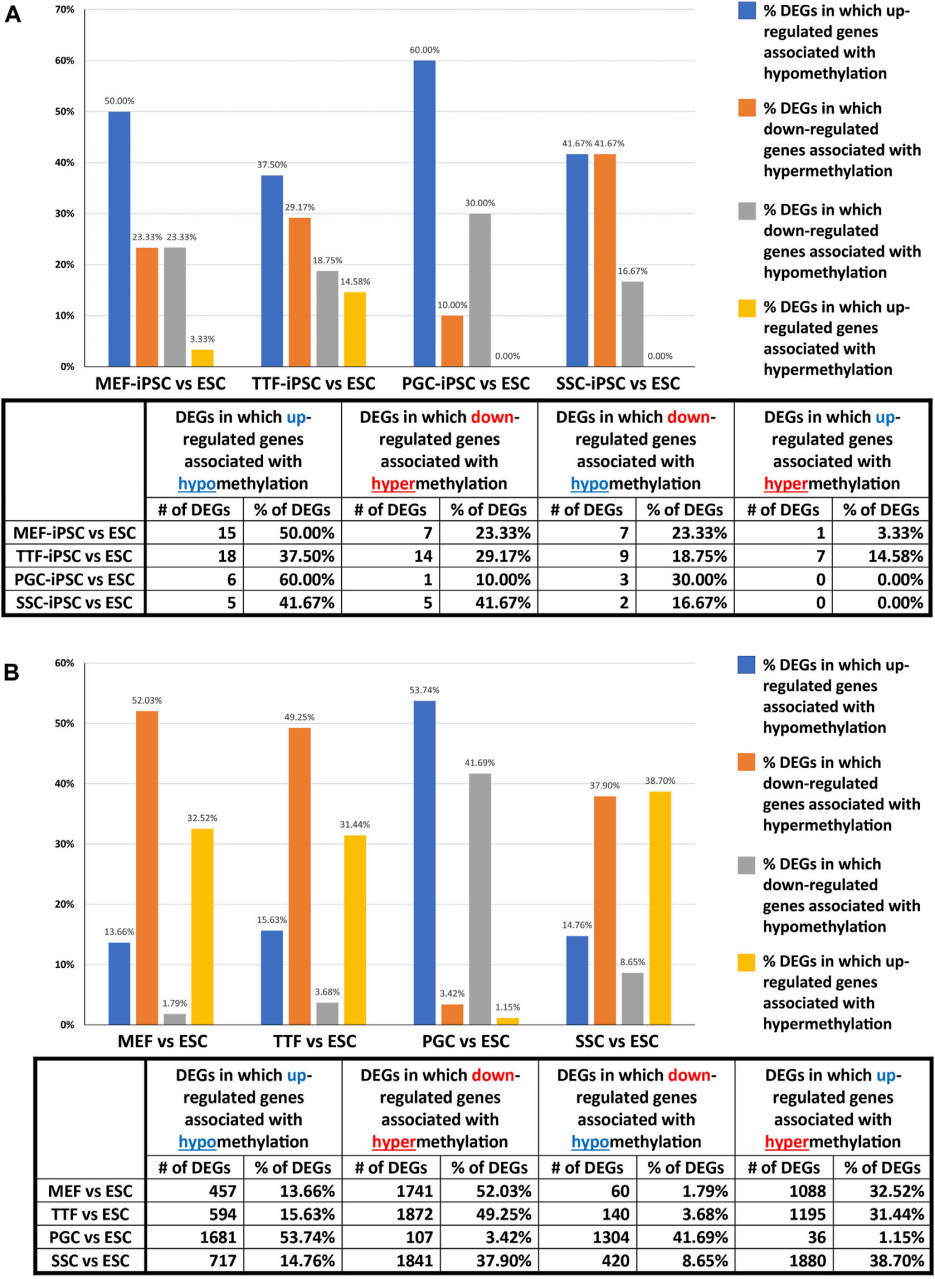
Correlation between DEGs and DMRs in promoter regions. Bar graphs illustrate the association between DEGs and increased (hyper) or decreased (hypo) DNA methylation levels in DMRs at promoter regions in **(A)** iPSC lines and **(B)** source cell types, relative to control ESCs. Promoter regions = 900-bp upstream—500-bp downstream of transcription start sites. Differential methylation = <30% where regions of ≥300 bp with ≤5 CpGs were excluded.

We further assessed the promoter-region DMR-containing DEGs that distinguished either the iPSC lines or the source cell types from the control ESC line to determine if there was concordance between hypermethylated DMRs and downregulated DEGs or hypomethylated DMRs and upregulated DEGs that persisted from the source cell types into the iPSC lines (Figure 5). Surprisingly, we were only able to correlate 16%–25% of the DEGs distinguishing the iPSC lines from the control ESC lines with the presence of promoter-region DMRs (Figure 4A). However, among those 16%–25% DEGs, we were able to match 67%–82% to DMRs with either hypomethylation associated with upregulated DEGs or hypermethylation associated with downregulated DEGs (Figure 5A). Similarly, we were able to correlate 51%–60% of the DEGs distinguishing the source cell types from the control ESC lines with the presence of promoter-region DMRs (Figure 4B), and, of those, 52%–66% showed either hypomethylated DMRs associated with upregulated DEGs or hypermethylated DMRs associated with downregulated DEGs (Figure 5B). A comparison of the promoter-region DMR-containing DEGs that distinguished the iPSCs from the control ESCs or the source differentiated cell types from the control ESCs revealed that a portion were common to both, including six distinguishing both the source MEFs and the MEF-iPSCs from the ESCs, another six distinguishing both the source TTFs and the TTF-iPSCs from the ESCs, as well as three distinguishing the source PGCs/M-prospermatogonia and the PGC-iPSCs from the ESCs and another three distinguishing the source SSCs and the SSC-iPSCs from the ESCs (Table 2). These results support the hypothesis that DNA methylation patterns in the starting cell type do indeed contribute, at least in part, to regulation of gene expression in iPSCs derived from that cell type. However, it also appears that genes can be predisposed to retain transcriptional memory even when differential DNA methylation that initially distinguished the differentiated source cell type from validated pluripotent ESCs becomes erased during the epigenetic reprogramming process associated with derivation of iPSCs from differentiated source cell types. This likely reflects persistent regulation by either epigenetic modifications other than DNA methylation (e.g., histone modifications), and/or lingering epigenetic programming at regulatory regions other than gene promoters (e.g., enhancers).

**TABLE 2:** DMRs in source cells and corresponding iPSCs.

# of DMRs groups and types of DMRs		Source cells vs. ESCs	iPSCs vs. ESCs	DMRs common to both source cells and iPSCs
MEFs/MEF-iPSCs	Hypo-DMRs^a^ associated with upregulated DEGs	457	15	3
	Hyper-DMRs^b^ associated with downregulated DEGs	1,741	7	3
TTFs/TTF-iPSCs	Hypo-DMRs associated with upregulated DEGs	594	18	3
	Hyper-DMRs associated with downregulated DEGs	1,872	14	3
PGCs/PGC-iPSCs	Hypo-DMRs associated with upregulated DEGs	1,681	6	3
	Hyper-DMRs associated with downregulated DEGs	107	1	0
SSCs/SSC-iPSCs	Hypo-DMRs associated with upregulated DEGs	717	5	2
	Hyper-DMRs associated with downregulated DEGs	1,841	5	1

^a^ Hypo-DMR, hypomethylated DMRs at promoter.

^b^ Hyper-DMR, hypermethylated DMRs at promoter.

**FIGURE 6:**
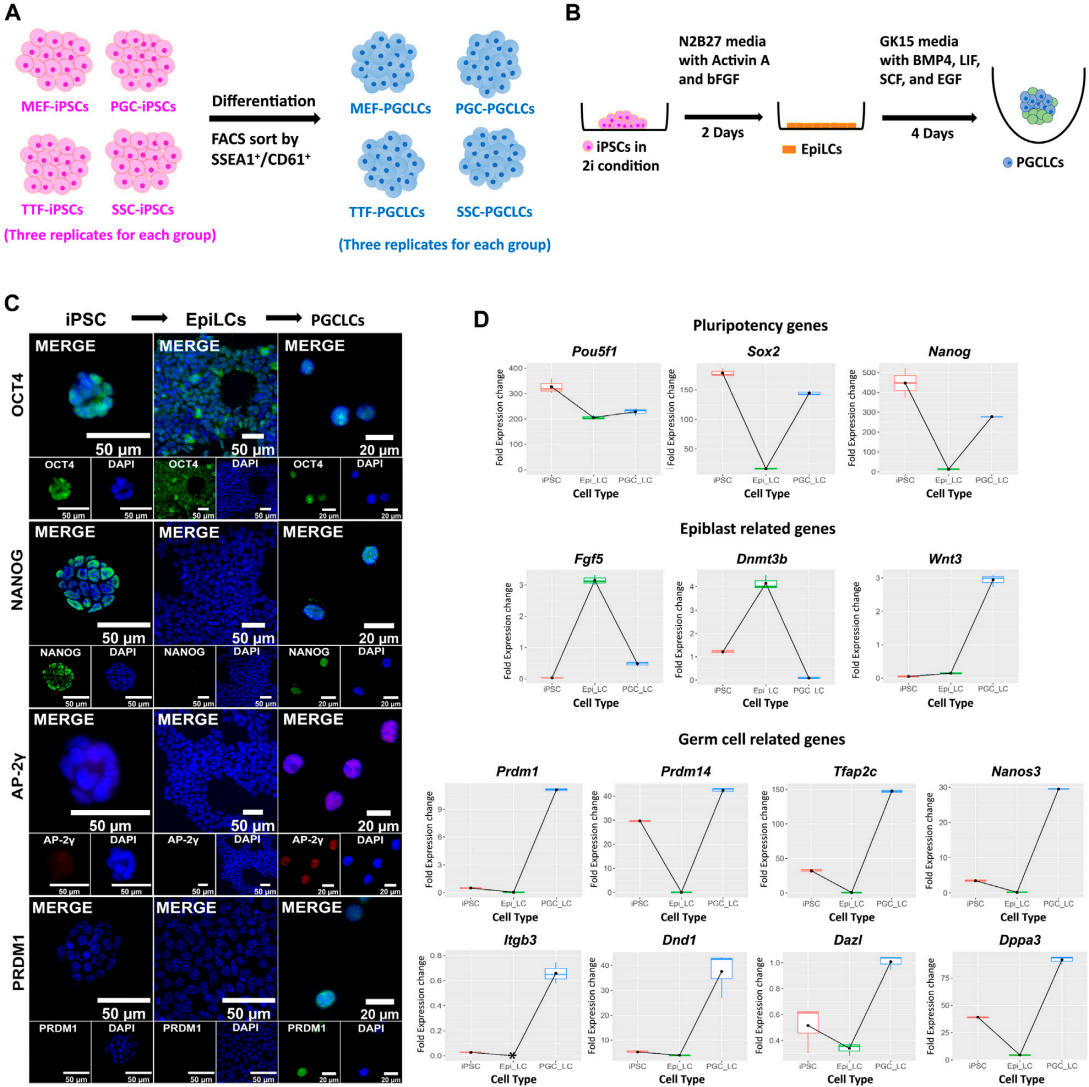
Validation of PGCLCs derived from iPSCs. **(A)** Experimental scheme of PGCLC differentiation from four sets of iPSCs. iPSC lines were induced to differentiate to form PGCLCs using a protocol published by the Saitou lab (Hayashi et al., 2011; Hayashi and Saitou, 2013a, 2013b). **(B)** A schematic shows the methodology for epiblast-like cell (EpiLC) and primordial germ cell-like cell (PGCLC) induction *in vitro*. Mouse iPSCs in 2i medium were induced to form EpiLCs for 2 days by addition of factors including Activin A and basic fibroblast growth factor (bFGF). EpiLCs were then induced to form PGCLCs in floating aggregates by addition of four additional growth factors—BMP4, EGF, LIF, and SCF for 4 days [figure modified from Saitou and Miyauchi (2016)]. **(C)** Immunocytochemistry staining for mouse pluripotency markers: OCT4 and NANOG, and germ cell markers: AP-2γ and PRDM1 during induction of PGCLCs from iPSCs through an EpiLC intermediary stage. **(D)** Gene expression profiles were measured by qRT-PCR for expression of pluripotency- (top row), epiblast- (second row), and germ cell-related (bottom two rows) genes during transitions from iPSCs to EpiLCs to PGCLCs, respectively. Fold expression levels were calculated relative to the control housekeeping gene, *Gusb*, using ΔCt methods.

Derivation of PGCLCs from iPSC lines. Derivation of PGCLCs from iPSCs was based on a two-phase process with the first phase involving induction of epiblast-like cells (EpiLCs) from the iPSCs, followed by a second phase involving induction of PGCLCs from the EpiLCs (Figure 6). We validated the resulting cell types by ICC staining and qRT-PCR for expression of pluripotency, epiblast and germ cell markers at the protein (Figure 6C) and RNA (Figure 6D; Supplementary Figure S2A) levels, respectively. WNT3 is a member of the WNT signaling pathway that is essential for development of PGCs (Ohinata et al., 2009). It is known to initiate expression in the epiblast (Kemp et al., 2005) to predispose the ability of epiblast cells to respond to BMP4 signaling to initiate progress toward germ cell fate (Ohinata et al., 2009). In our *in vitro*-derived cell types, the *Wnt3* gene showed low level expression in EpiLCs and then dramatically increased expression in PGCLCs (Figure 6D). Additionally, all eight germ-cell markers that we tested (*Dazl*, *Dnd1*, *Dppa3*, *Itgb3*, *Nanos3*, *Prdm1*, *Prdm14*, and *Tfap2c*) showed high expression in PGCLCs relative to iPSCs or EpiLCs (Figure 6D), except *Prdm14* which also showed high expression in iPSCs. Taken together, this data showing transcript levels for 14 key cell-type specific marker genes, in conjunction with our comprehensive assessment of epigenetic programming and gene expression profiles in the iPSCs and PGCLCs, validates the successful induction of transitions *in vitro*—from iPSCs to EpiLCs to PGCLCs—that mimick those that occur between corresponding cell types *in vivo*—from inner cell mass cells to epiblast cells to PGCs. This supports our interpretation that the disappearance of epigenetic/transcriptomic memory detectable in the iPSCs but not in the PGCLCs occurs coincident with germline epigenetic reprogramming similar to that which occurs *in vivo*. However, assessment of the efficiency of differentiation of iPSCs to form PGCLCs revealed differences among the PGCLCs induced from the different iPSC groups, varying from a low of 10.1% for TTF-PGCLCs to 15.0% for SSC-PGCLCs, 16.7% for MEF-PGCLCs and 18.1% for PGC-PGCLCs (Supplementary Figure S2B). Although these differences were not significant. They are consistent with the notion that iPSCs derived from earlier stage cell types, be they somatic or germ cell types, are able to form PGCLCs at least slightly more efficiently than iPSCs derived from later stage cell types.

**FIGURE 7:**
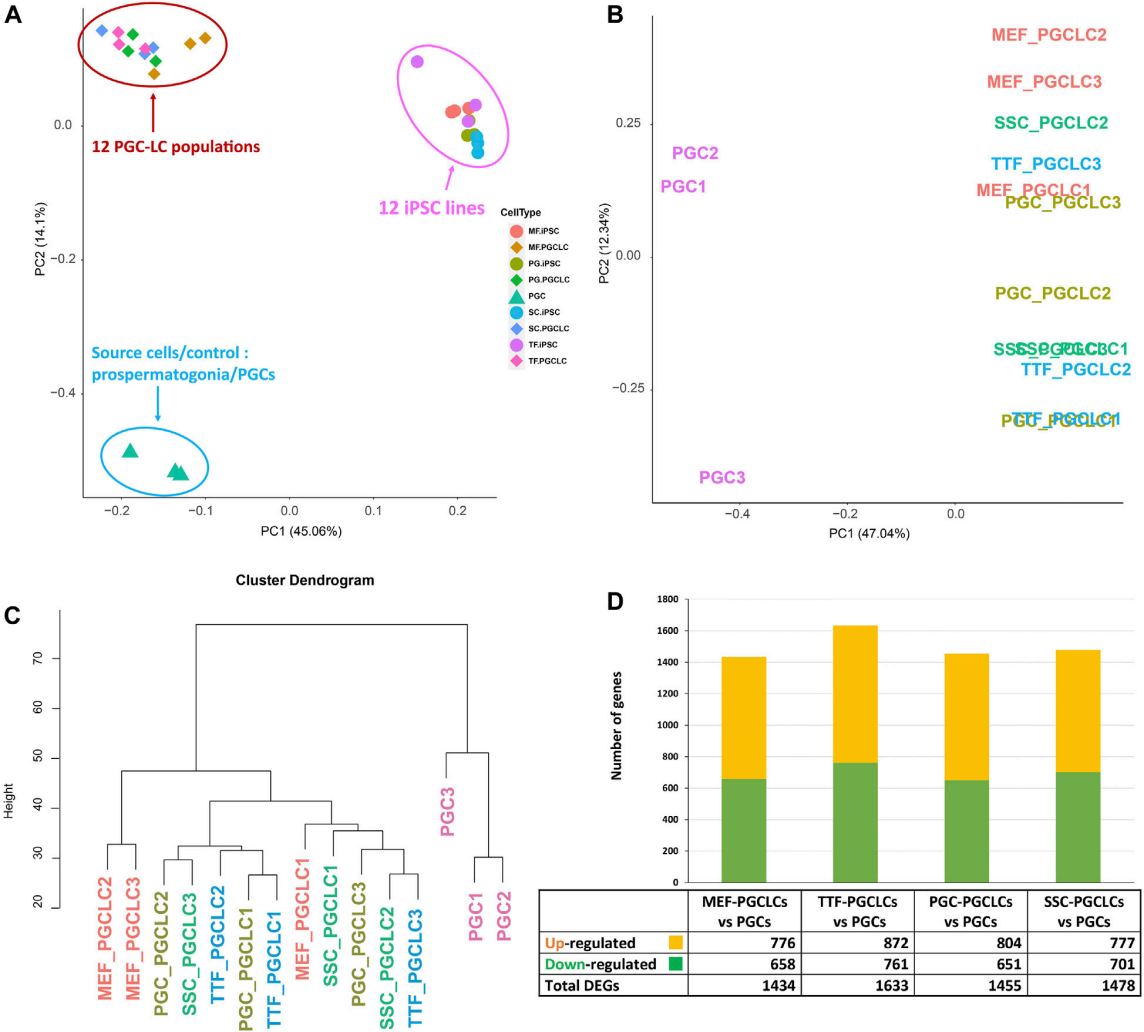
RNA-seq transcriptome analysis of PGCLCs. **(A)** A PCA of RNA-seq-based transcription profiles for three replicates of each of four PGCLC populations derived from each of four different groups of iPSCs. Control data represent combined values from three replicates of endogenous E13.5 PGCs/M-prospermatogonia. Samples are clustered based on transcription profiles. **(B)** A PCA analysis focused strictly on transcription profiles of the 12 PGCLC replicates representing the 4 PGCLC groups, plus the 3 endogenous E13.5 PGC/M-prospermatogonia replicates. **(C)** A hierarchical clustering dendrogram of the same 12 PGCLC and the 3 endogenous E13.5 PGC/M-prospermatogonia samples in which transcription profiles were generated based on 1-Pearson’s correlation distance. **(D)** Quantification of bulk RNA-seq data revealed DEGs in each group of PGCLCs compared to the control endogenous E13.5 PGCs/M-prospermatogonia. Differential expression = ≥1.5-fold change, FDR <5%, *p* < 0.05, and *n* = 3 in each group.

PGCLC populations do not retain significant transcriptional memory during the iPSC to PGCLC transition. Each group of PGCLCs was then assessed for gene expression patterns by RNA-seq and the results were compared to RNA-seq data from the control endogenous PGCs/M-prospermatogonia (Figure 7). The PCA analysis in Figure 7A revealed three well-separated clusters. As expected, all PGCLC populations grouped together separately from the iPSCs. The third cluster represents the endogenous E13.5 PGCs/M-prospermatogonia. However, as shown in Figures 7A–C, none of the PGCLC populations grouped together with the control endogenous E13.5 PGCs/M-prospermatogonia. This is likely because the PGCLCs more closely resemble an earlier stage in germ cell development. Indeed, previous reports have suggested that day 4 PGCLCs more closely resemble endogenous PGCs at an early migratory stage (equivalent to endogenous E8.5-E9.5) rather than PGCs/M-prospermatogonia at E13.5 (Seisenberger et al., 2012; Kobayashi et al., 2013; Shirane et al., 2016; Ohta et al., 2017). Therefore, it is not surprising that the PGCLCs we derived from each group of iPSCs clustered separately from the control E13.5 PGCs/M-prospermatogonia in the PCA.

To further examine potential differences among the 12 PGCLC samples (three replicates derived from each of the four iPSC groups) and the three endogenous control E13.5 PGCs/M-prospermatogonia replicates, we replotted only the PGCLC and control E13.5 PGCs/M-prospermatogonia data (Figure 7B). This refined PCA plot plus hierarchical clustering of transcriptome data revealed general differences between the PGCLCs and E13.5 PGCs/M-prospermatogonia (Figures 7B, C). Interestingly, in the PCA of gene expression data in Figure 7B, all but one of the replicate samples of PGCLCs derived from each group of iPSCs clustered closely together. Importantly, each set of replicates (replicate sets #1, #2 and #3) of PGCLCs were derived from iPSCs at the same passage number. Thus, the #1 replicates of PGCLCs from the four different groups of iPSCs were all derived from iPSCs at passage 15, while the #2 replicates were all derived from iPSCs at passage 17 and the #3 replicates were all derived from iPSCs at passage 19. Hayashi and Saitou (2013a, 2013b) reported that PGCLCs derived from iPSCs at different passage numbers will vary. Moreover, the pattern of DEGs (Figure 7D) appears to reflect the efficiency of PGCLC differentiation for each group (Figure 6B). TTF-PGCLCs, which showed the highest amount of DEGs among all four PGCLC groups when compared to endogenous E13.5 PGCs/M-prospermatogonia, showed the lowest efficiency of PGCLC derivation among all PGCLC groups. On the other hand, MEF-PGCLCs, PGC-PGCLCs, and SSC-PGCLCs, which showed similar numbers of DEGs relative to the endogenous E13.5 PGCs/M-prospermatogonia, also showed similar efficiencies of PGCLC derivation, all of which were higher than the efficiency with which the TTF-PGCLCs were derived. While this result may suggest a minor indication of developmental memory between the different iPSC groups and the corresponding PGCLC groups, we did not detect evidence of transcriptional memory lingering during the iPSC to PGCLC transition.

To determine if PGCLCs retain transcriptional memory from the iPSC line from which they were derived, which might also reflect transcriptional memory from the corresponding source cells used to generate the corresponding line of iPSCs, we compared the list of DEGs between each group of PGCLCs and the control endogenous E13.5 PGCs/M-prospermatogonia with the list of DEGs between the corresponding groups of iPSCs and their source cell types (Figure 7). For example, we compared DEGs found between MEF-PGCLCs and E13.5 PGCs/M-prospermatogonia with the list of DEGs we previously found between MEF-iPSCs and the reference ESCs. In this way, we sought to determine if DEGs distinguishing each group of PGCLCs may have been carried over from DEGs already present in the corresponding iPSCs due to epigenetic memory from the source cell type from which the iPSCs were derived.

As shown in Table 3 and Supplementary Table S4, a large majority of the apparent transcriptional memory persisting in each group of iPSCs from their source cell types was not carried forward to the corresponding groups of PGCLCs. Thus, we found that of 77 DEGs (either up- or downregulated) in MEF-iPSCs that were retained from the source differentiated cell type (MEFs), only 2 were carried on to the corresponding MEF-PGCLCs and therefore manifest as DEGs between the MEF-PGCLCs and the control endogenous E13.5 PGCs/M-prospermatogonia. Similarly, we detected 113, 26, and 36 source cell-type specific DEGs in TTF-iPSCs, SSC-iPSCs and PGC-iPSCs, respectively, and, of those, only 9, 1 and 0 persisted into the corresponding PGCLCs when those populations were compared to endogenous E13.5 PGCs/M-prospermatogonia. This overall lack of transcriptional memory persisting into PGCLCs appears to directly reflect the more extensive epigenetic reprogramming that normally occurs during germline development, which PGCLCs have initiated. Thus, just as endogenous PGCs reach a unique nadir of hypomethylation by more extensively erasing inherited DNA methylation than any other cell type, including pluripotent cells in the preimplantation embryo, PGCLCs appear to more effectively erase inherited DNA methylation than do iPSCs.

**TABLE 3:** DEGs in source cells and corresponding PGCLCs.

# of DEGs groups and types of DEGs		Common to source cells and iPSCs vs. ESCs	PGCLCs vs. PGCs>	DEGs that are common to both
MEF	Upregulated	19	776	1
	Downregulated	58	658	1
TTF	Upregulated	18	872	1
	Downregulated	95	761	8
PGC	Upregulated	22	804	0
	Downregulated	14	651	0
SSC	Upregulated	8	777	1
	Downregulated	18	701	0

**FIGURE 8:**
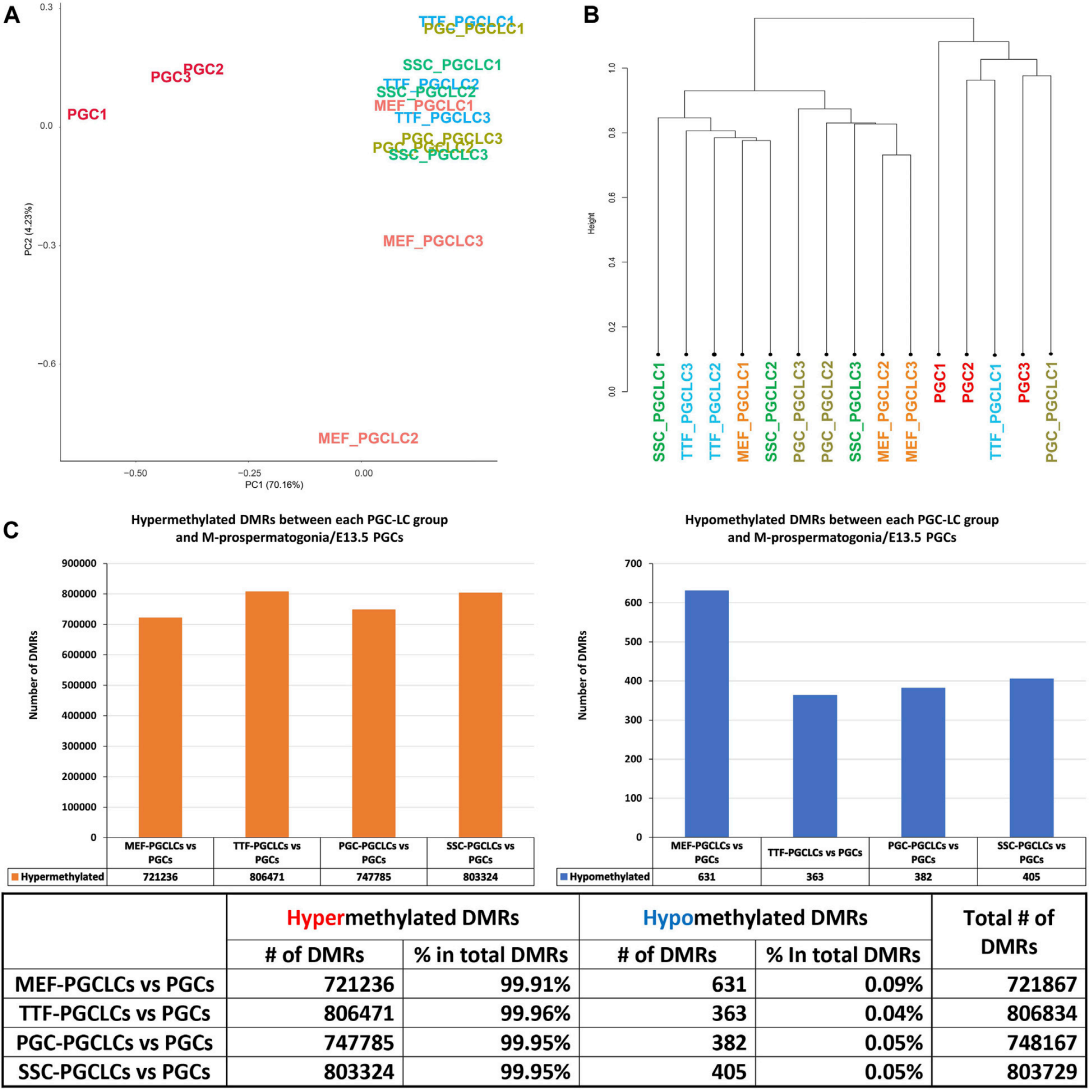
Analysis of DNA methylation profiles in PGCLC populations using whole-genome bisulfite sequencing (WGBS). **(A)** A PCA of CpG DNA methylation profiles in three replicates each of the four groups of PGCLC populations and 1 control group of endogenous E13.5 PGCs/M-prospermatogonia. **(B)** Hierarchical clustering of CpG DNA methylation profiles in the 12 PGCLC populations (three replicates each from the four groups of PGCLCs) and three replicates of control endogenous E13.5 PGCs/M-prospermatogonia based on 1-Pearson’s correlation distance. **(C)** Quantification of DMRs in each group of PGCLCs compared to control endogenous E13.5 PGCs/M-prospermatogonia. The bar graphs illustrate the total number of hypomethylated and hypermethylated DMRs found in each comparison. Differential methylation differences <30% and regions of ≥300 bp with ≤5 CpGs were excluded; *n* = 3 in each group.

PGCLC populations do not retain significant epigenetic memory during the iPSC to PGCLC transition. WGBS analysis of triplicate samples of PGCLCs differentiated from each set of iPSCs originally derived from each different source cell type was used to determine how closely the *in vitro*-derived PGCLCs resembled control endogenous E13.5 PGCs/M-prospermatogonia, and to what extent any epigenetic memory detectable in each set of iPSCs was retained following differentiation of those iPSCs to form PGCLCs (Figure 8). Thus, we analyzed DNA methylation patterns in the same three replicates of PGCLCs induced from each of the four different types of iPSCs that were analyzed for gene expression as described above. The PCA and hierarchical clustering analyses of our WGBS data shows that each set of PGCLCs clustered close to one another with the exception of two apparent outliers—MEF-PGCLC replicates 2 and 3 (Figures 8A, B). In addition, the four groups of PGCLCs consistently showed very similar patterns of hypermethylated and hypomethylated DMRs (Figure 8C). When compared to the control endogenous E13.5 PGCs/M-prospermatogonia, nearly all DMRs (>99%) detected in all four groups of PGCLCs were hypermethylated (Figure 8C). Once again, this would appear to reflect the fact that day 4 PGCLCs more closely resemble endogenous PGCs at an early developmental stage (E8.5-E9.5) which have not progressed as far into germline-specific epigenetic reprogramming as the endogenous E13.5 PGCs/M-prospermatogonia (Seki et al., 2005; Guibert et al., 2012; Seisenberger et al., 2012).

**FIGURE 9:**
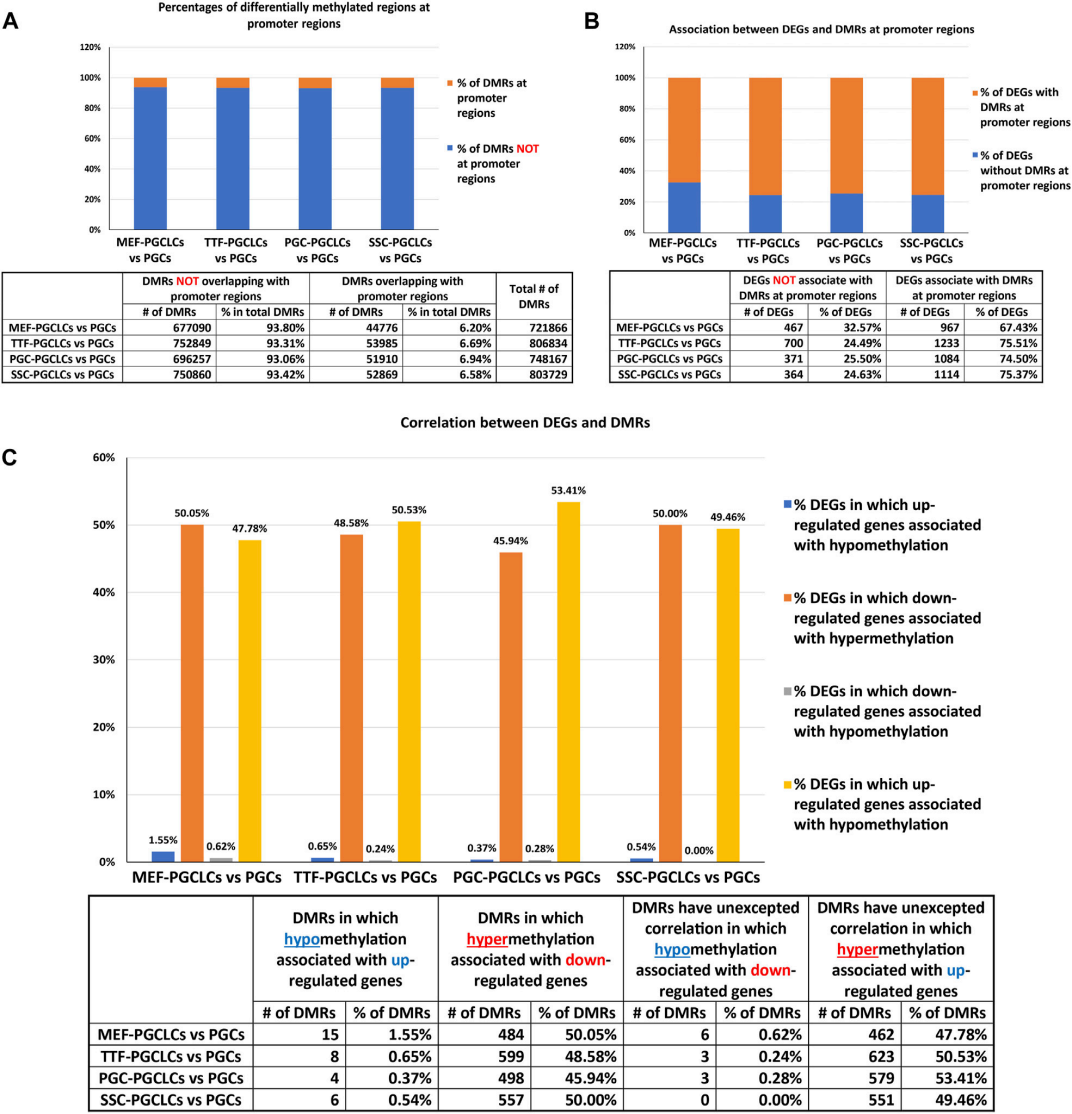
Annotation of differentially methylated regions. **(A)** Bar graphs show percentages of DMRs occurring in promoter regions in PGCLC populations when compared to endogenous E13.5 PGCs/M-prospermatogonia. **(B)** Bar graphs display percentages of DEGs that have DMRs in their promoter regions in PGCLC populations relative to E13.5 PGCs/M-prospermatogonia. **(C)** Bar graphs illustrate the association between DEGs and increased (hyper) or decreased (hypo) DNA methylation levels in DMRs at promoter regions in PGCLC groups relative to E13.5 PGCs/M-prospermatogonia. (Promoter regions = 900-bp upstream—500-bp downstream of the transcription start sites. Differential methylation = a <30% difference in DNA methylation level within regions of ≥300 bp with ≤5 CpGs were excluded; biological replicates—*n* = 3/group.)

Relationship between DMR and DEG patterns in PGCLCs. To determine the extent to which the limited variation in gene expression patterns we detected in the form of DEGs among the different populations of PGCLCs correlated with differential epigenetic programming in the form of DMRs, we mined our WGBS data for the presence of DMRs in promoter regions of DEGs. In general, fewer than 10% of DMRs distinguishing the PGCLC populations derived from all four sets of iPSCs occurred in gene promoter regions when compared to the control endogenous E13.5 PGCs/M-prospermatogonia (Figure 9A), reflecting the overall absence of epigenetic and transcriptomic memory in the PGCLCs. However, among the relatively low level of DEGs that were detected in the PGCLCs when compared to the endogenous E13.5 PGCs/M-prospermatogonia, two-thirds or more were found to have DMRs in their promoter regions (Figure 9B). This indicates that the very low level of transcriptional memory lingering in PGCLCs that appears to reflect cell-type specific transcriptomes from the source cell types that gave rise to the iPSCs from which the PGCLCs were derived is correlated with a correspondingly low level of retained epigenetic memory.

We next examined the extent to which patterns of differential up- vs downregulation of gene expression correlated with expected corresponding differential hypo-versus hyper- DNA methylation, respectively. We found that about half of the downregulated DEGs showed the expected correlation with promoter region hypermethylated DMRs (Figure 9C). In contrast, however, very few DEGs that were upregulated in the PGCLC groups compared to endogenous E13.5 PGCs/M-prospermatogonia displayed hypomethylated promoter region DMRs (Figure 9C). This likely reflects the uniquely low level of genome-wide DNA methylation present in the reference control cell type used for this analysis—E13.5 PGCs/M-prospermatogonia. Thus, in this case, other epigenetic regulatory parameters—such as histone modifications, along with transcription factors, are likely to be the predominant determinants of gene expression levels, and, hence, DEGs, rather than differential DNA methylation. Importantly, we did not observe notable differences in this regard among the PGCLC populations derived from the different groups of iPSCs, indicating that this was not impacted by epigenetic memory.

In summary, while we did detect apparent epigenetic memory associated with DEGs found in each group of source differentiated cell types and the corresponding groups of iPSCs derived from those source cell types, we detected very little epigenetic memory associated with DEGs found in each group of PGCLCs that appeared to be inherited from the corresponding source group of iPSCs (Table 4). This could be explained by either or both of two possibilities: 1) because during natural development *in vivo* germline reprogramming ultimately leads to formation of the epigenetic ground state, recapitulation of even the first portion of that reprogramming during the transition from iPSCs to PGCLCs may lead to substantial erasure of most, if not all inherited epigenetic programming, thereby precluding transmission of epigenetic memory during this transition *in vitro*, and/or 2) prolonged culture in general, regardless of what transition in cell fate may be induced, may promote a general loss of epigenetic memory (Polo et al., 2010). Taken together, our data reveals that epigenetic memory detected in iPSCs is largely erased in PGCLCs derived from those iPSCs.

**TABLE 4:** Source cell-type specific epigenetic memory transmitted from iPSCs to corresponding PGCLCs.

Source cells	Correlation between DEGs and DMRs	Epigenetic memory inherited from source cells and retained in iPSCs	Epigenetic memory inherited from iPSCs and retained in PGCLCs	Note
MEF	Hypomethylation associated with upregulated genes	*Arhgef2*, *Frmd6*, *Zfp800*	N/A^a^	*Arhgef2* was found in PGCLCs but associated with hypermethylation instead of hypomethylation
	Hypermethylation associated with downregulated genes	*Commd1*, *Ccnb1ip1*, *Nek2*	N/A	
TTF	Hypomethylation associated with upregulated genes	*Arhgef2*, *Arrb1*, *Hpcal1*	N/A	
	Hypermethylation associated with downregulated genes	*Jam2*, *Mta1*, *Trmt1*	N/A	
PGC	Hypomethylation associated with upregulated genes	*Hpcal1*, *Klhdc2*, *Uty*	N/A	*Uty* was found in PGCLCs but associated with hypermethylation instead of hypomethylation
	Hypermethylation associated with downregulated genes	N/A	N/A	
SSC	Hypomethylation associated with upregulated genes	*Egln3*, *Uty*	*N/A*	*Egln3* was found in PGCLCs but associated with hypermethylation instead of hypomethylation
	Hypermethylation associated with downregulated genes	*Crxos*	*N/A*	

^a^ No genes found.

## Discussion

The ability to derive functional gametes *in vitro* has been a long-standing objective to facilitate a potentially powerful strategy for treatment of infertility (Hayashi et al., 2011; Gu et al., 2012; Cai et al., 2013; Fattahi et al., 2017; Seita et al., 2023). The discovery that differentiated somatic cell types can be reprogrammed to form iPSCs (Takahashi and Yamanaka, 2006) opened the possibility to generate patient-specific pluripotent cells from which germline cell types, and ultimately gametes, can be derived for use with assisted reproductive technologies to generate biological offspring of otherwise infertile adults (Ishikura et al., 2016; Ishikura et al., 2021). However, for this approach to be applicable in a clinical setting, it is critical that the gametes resulting from this process of *in vitro* gametogenesis be as pristine as possible, particularly with respect to proper epigenetic programming that will direct normal gene expression during development of the resulting offspring.

Shortly after the discovery of iPS reprogramming methodology it was reported that iPSCs tended to retain epigenetic and transcriptional memory of the source differentiated cell types from which they were derived (Mikkelsen et al., 2008; Kim et al., 2010; Polo et al., 2010; Kim et al., 2011; Phetfong et al., 2016). This represents a potential concern that could limit the clinical utility of iPSC-based *in vitro* gametogenesis for treatment of infertility. Normal mammalian development includes two major phases of epigenetic reprogramming—one, termed embryonic reprogramming, that occurs in the preimplantation embryo during development of the epiblast, and a second, termed germline reprogramming, that occurs as PGCs develop and give rise to either oogonia in females or prospermatogonia in males (Morgan et al., 2005; Surani et al., 2007; Saitou et al., 2012; von Meyenn and Reik, 2015; Saitou and Miyauchi, 2016). The derivation of iPSCs from differentiated somatic cells should recapitulate embryonic reprogramming, whereas that of germ cells from iPSCs should recapitulate germline reprogramming. While it was previously demonstrated that the derivation of iPSCs is often accompanied by epigenetic and transcriptional memory, it was not previously well established to what extent the derivation of germline cells from iPSCs is or is not also associated with epigenetic or transcriptional memory.

Here we assessed the potential of different differentiated somatic and germ cell types to undergo iPSC reprogramming, and of the resulting iPSCs to then undergo PGCLC differentiation. We found that germ cell types underwent iPSC reprogramming more rapidly, but that somatic cell types underwent iPSC reprogramming more efficiently. However, this may simply reflect initial compatibility between each cell type and the culture medium that was used for iPSC reprogramming which may have supported growth and maintenance of somatic and germ cell types differentially. With respect to germline reprogramming, we found that iPSC lines derived from fetal source cell types—PGCs/M-prospermatogonia and MEFs showed slightly greater efficiency than iPSC lines derived from postnatal source cell types—SSCs and TTFs, but these differences were not large.

We analyzed gene expression patterns in iPSCs derived from four distinct source cell types, as well as in each of the corresponding four source cell types. We detected gene expression differences among the four sets of iPSCs that clearly reflected differences initially present in the source cell types, indicative of transcriptional memory persisting during the iPS reprogramming process as previously reported (Chin et al., 2009; Kim et al., 2010; Polo et al., 2010). We then analyzed DNA methylation patterns in the four sets of iPSCs and the four source cell types from which they were derived and found corresponding epigenetic programming persisting from the source cell types into the resulting sets of iPSCs, indicative of lingering epigenetic memory. A significant portion of the differential gene expression we detected between the derived iPSC lines and the control ESC line could be correlated with differential DNA methylation in promoter regions, with hypomethylated DMRs aligning with upregulated DEGs and hypermethylated DMRs aligning with downregulated DEGs. However, we also observed the opposite pattern in several cases as well, with hypomethylated DMRs occurring in promoter regions of downregulated DEGs and hypermethylated DMRs occurring in promoter regions of upregulated DEGs. In this respect it is important to note that DNA methylation is only one of several different epigenetic parameters, and promoters are only one type of regulatory region that controls gene expression. It is likely that the examples of apparent discordance we observed between hyper- and hypo-DNA methylation and up- and downregulation of gene expression, respectively, or the examples where we were not able to correlate DEGs with the presence of DMRs in either direction, can be explained by other types of epigenetic programming and/or transcription factor binding function in other relevant regulatory regions, such as enhancers. This notion was further evidenced by our observation that during iPSC reprogramming, many genes that showed both differential expression and differential DNA methylation among the source differentiated cell types continued to show differential expression even in the absence of differential DNA methylation in the iPSC lines derived from each source. In these cases, the lingering differential gene expression cannot be explained by differential promoter region DNA methylation, but likely reflects either other types of differential epigenetic programming in the promoter regions—such as activating versus repressive histone modifications—or differential DNA methylation in non-promoter regulatory regions such as enhancers.

We next induced each set of iPSCs to differentiate first into EpiLCs and then into PGCLCs. Assessment of multiple markers validated these transitions in cell fate. We compared gene expression and DNA methylation patterns in the resulting sets of PGCLCs with those in endogenous male PGCs/M-prospermatogonia recovered from fetuses at E13.5. As previously reported, we found that PGCLCs derived from male PSCs differed somewhat from endogenous male PGCs/M-prospermatogonia, consistent with the suggestion that PGCLCs most closely resemble earlier, migrating PGCs rather than PGCs that have colonized the developing testis to give rise to M-prospermatogonia (Shirane et al., 2016; Ohta et al., 2017). However, our assessment of gene expression patterns in each set of PGCLCs showed much less variation than we observed among the gene expression patterns in each set of iPSCs originally derived from each of the different source cell types. More importantly, to the extent that we did observe differences in gene expression patterns between each set of PGCLCs and the control endogenous PGCs/M-prospermatogonia, there was very little evidence that this reflected transcriptional memory persisting from the original source cell types from which each set of iPSCs was derived. This was further corroborated by our analysis of DNA methylation patterns in the four sets of PGCLCs. Once again, we did detect differences in epigenetic programming between the PGCLCs and the control PGCs/M-prospermatogonia indicative of the former more closely resembling migrating PGCs than gonadal PGCs. However, as with the gene expression patterns in each set of PGCLCs, we detected less variation in DNA methylation patterns among the four sets of PGCLCs than among the four sets of iPSCs, and very little evidence that the variation we did detect emanated from epigenetic memory persisting from the original source cell types from which the different sets of iPSCs were derived.

It has been reported that lingering epigenetic/transcriptomic memory normally disappears with extended culture (Chin et al., 2009; Polo et al., 2010), and derivation of PGCLCs from iPSCs requires additional culture beyond that required for initial derivation of iPSCs. Thus, it could be suggested that the disappearance of epigenetic/transcriptomic memory we observed in PGCLCs was solely due to the extended culture period required to derive PGCLCs from iPSCs. However, we suggest the disappearance of epigenetic/transcriptomic memory we observed during the iPSC to PGCLC transition was due to the more extensive reprogramming that occurs during this transition. This is because the initial derivation of iPSCs from starting differentiated source cell types required 21–30 days and 13–14 passages in culture and was still not sufficient to erase lingering epigenetic/transcriptomic memory from the resulting iPSCs. By comparison, the derivation of PGCLCs from iPSCs was accomplished in only 7–10 additional days and 2–3 additional passages in culture and did result in nearly complete erasure of all epigenetic/transcriptomic memory. Clearly, this final erasure of lingering epigenetic/transcriptomic memory occurred at an accelerated rate, which we ascribe to enhanced “germline-like” reprogramming.

Taken together, these results indicate that despite the persistence of transcriptional and epigenetic memory during the derivation of iPSCs from various differentiated source cell types, a large majority of this memory is erased when iPSCs are then induced to undergo germline reprogramming to form PGCLCs. Interestingly, it has been reported that epigenetic programming in PGCLCs derived *in vitro* from PSCs resembles that in endogenous migratory PGCs *in vivo* more so than that in later PGCs that have colonized the gonads (Ohta et al., 2017). Indeed, induction of further germline development of PGCLCs *in vitro* to what are termed “expanded PGCLCs” has been shown to promote even more extensive erasure of epigenetic programming as these cells reach the “epigenetic ground state” found uniquely in fetal germ cells (Hajkova, 2011; Ohta et al., 2017). We suggest that this reflects a distinction between the germline and iPS reprogramming processes, in that germline reprogramming more thoroughly erases epigenetic and, hence, transcriptomic memory in cells initially derived *in vitro* from differentiated cell types than does iPS reprogramming. In turn, these findings support the potential clinical utility of *in vitro*-derived gametes as a treatment for infertility given that they suggest that this process will preclude lingering abnormal epigenetic memory in the resulting gametes.

## Data Availability

The data presented in the study, except the SSC RNA-seq, are deposited in the Gene Expression Omnibus (GEO) under the accession number GSE254465. The RNA-seq data for ID4-eGFP^Bright^ SSC presented in this study can also be found in the NCBI GEO database, with the accession number GSE131653.
