# Supplementary material for: Source cell-type epigenetic memory persists in induced pluripotent cells but is lost in subsequently derived germline cells
Source: Front Cell Dev Biol. 2024 Feb 12;12:1306530. doi: 10.3389/fcell.2024.1306530 (PMC10895008; doi:10.3389/fcell.2024.1306530)
Supplement: Supplementary file 1 [file DataSheet2.PDF]

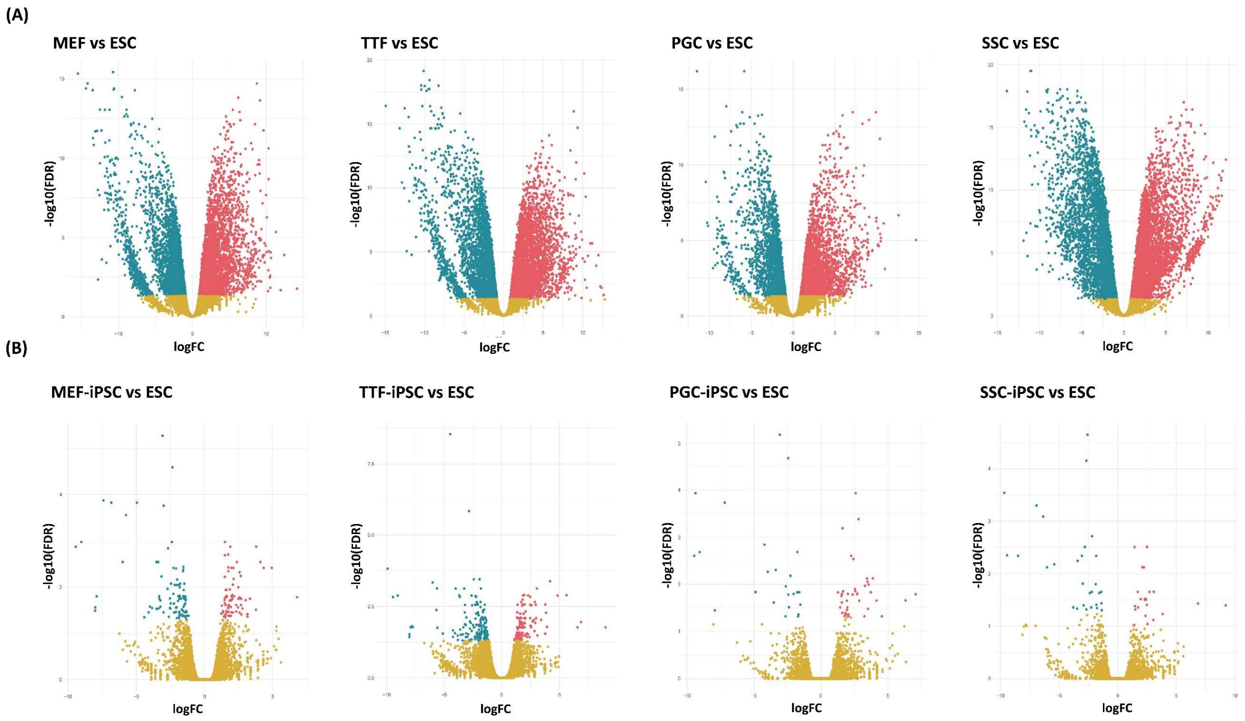

Figure S1. Visualization of RNA-seq results with volcano plots. (A) Each group of source cells versus control ESC lines. (B) Each group of iPSC lines versus control ESC lines. The negative log of FDR (base 10) is plotted on the Y-axis, and the log of the FC (base 2) is plotted on the X-axis. The red points on the plot represent up-regulated genes and the blue points represent down-regulated genes that are significantly differently expressed, the light brown points represent genes are not significantly differently expressed in either source cells or iPSCs when compared to ESCs. Differential gene expression = ≥1.5x fold change, FDR < 5%, and n=3 in each group.

(A)

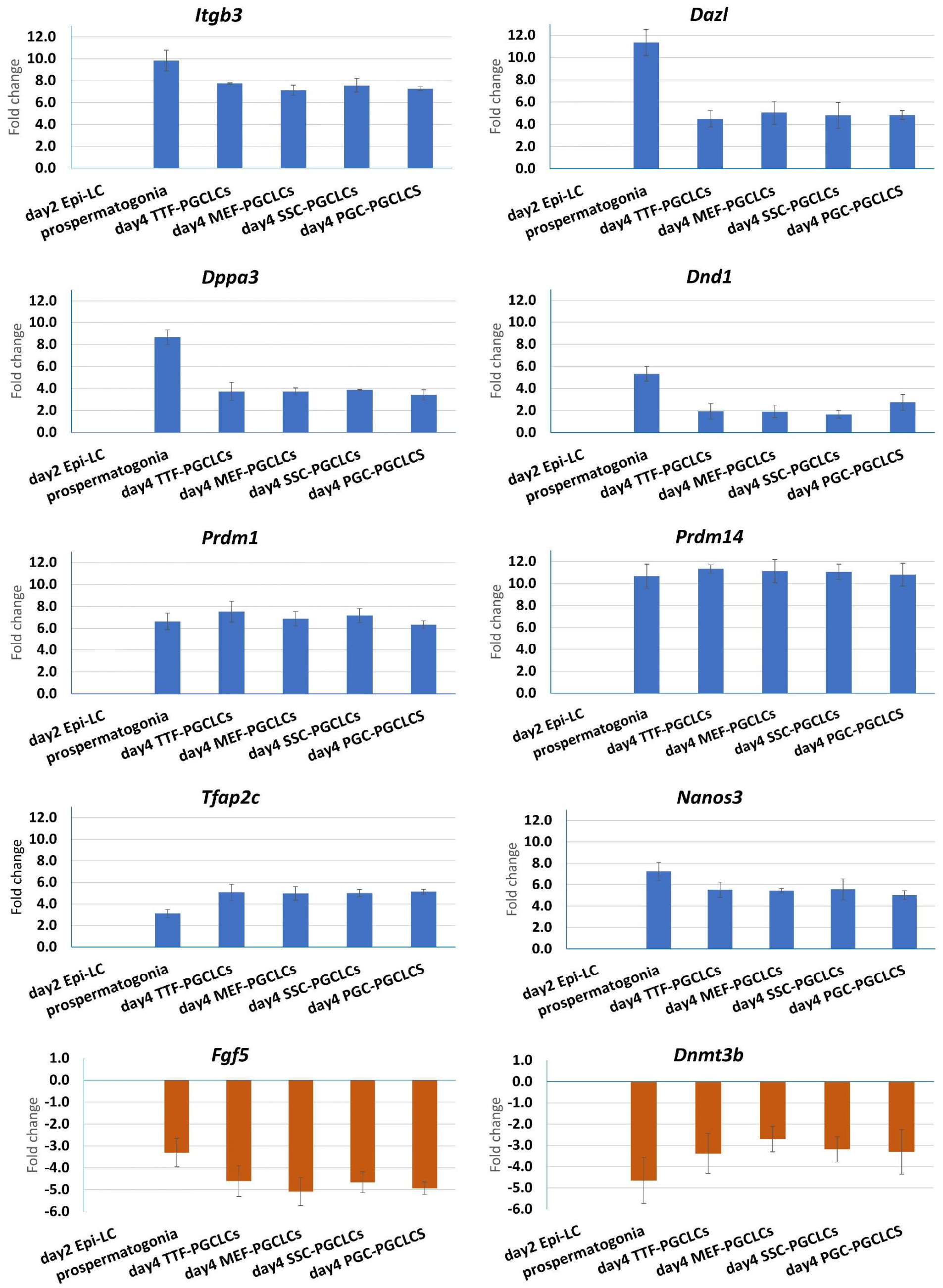

(B)

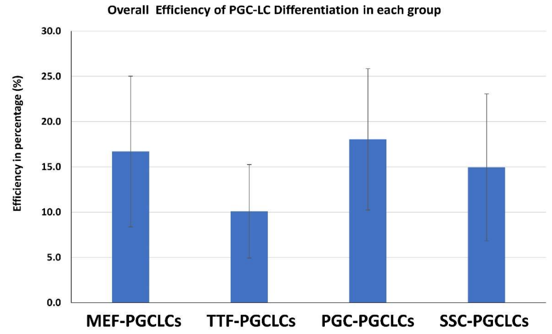

Figure S2. Gene expression profiles and differentiation efficiency in day 4 PGCLCs derived

from four groups of iPSC lines. (A) Quantification of expression of epiblast and germ cell marker genes in MEF-PGCLCs, TTF-PGCLCS, PGC-PGCLCs and SSC-PGCLCS were measured by qRT-PCR. For each gene, the ΔCT was calculated from the CT value of the control housekeeping gene Gusβ. Then, the ΔΔCT from the CT value from the negative control EpiLCs was calculated and set at zero. Fold change is shown on the Y axes in $\log_2$ scale. n=2 for each sample. (B) Differentiation efficiency of MEF-PGCLCs, TTF-PGCLCs, PGC-PGCLCs, and SSC-PGCLCs induced from MEF-iPSCs, TTF-iPSCs, PGC-iPSCs, and SSC-iPSCs, respectively. The bar graph illustrates the average efficiency for each PGCLC group. n=5 for each group. Efficiency was calculated as the percentage of the INTEGRIN β3+/SSEA-1+ double positive cells within aggregates collected at day 4 of the PGCLC differentiation process. The significance was determined by one-way ANOVA followed by Tukey's test, and there was no statistically significant difference between each group.
