# Supplementary material for: Source cell-type epigenetic memory persists in induced pluripotent cells but is lost in subsequently derived germline cells
Source: Front Cell Dev Biol. 2024 Feb 12;12:1306530. doi: 10.3389/fcell.2024.1306530 (PMC10895008; doi:10.3389/fcell.2024.1306530)
Supplement: Supplementary file 2 [file DataSheet1.PDF]

Table S1. Sequences of qRT-PCR primers used for iPSC characterization

Gene Name	Forward primer sequence	Reverse primer sequence
Pou5f1	ACATCGCCAATCAGCTTGG	AGAACCATACTCGAACCACATCC
Sox2	TAGAGCTAGACTCCGGGCGATGA	TTGCCTTAAACAAGACCACGAAA
Nanog	GCTCCGCTCCATAACTTCGG	TTGTGGGGTGCTAAAATGCG
Klf4	GCACACCTGCGAACTCACAC	CCGTCCCAGTCACAGTGGTAA
Gapdh	CTCGTCCCGTAGACAAAATGG	ATCTCCACTTTGCCACTGCAA

Table S2. Sequences of qRT-PCR primers used for PGCLC validation

Gene Name	Forward primer sequence	Reverse primer sequence
Pou5f1	ACATCGCCAATCAGCTTGG	AGAACCATACTCGAACCACATCC
Sox2	TAGAGCTAGACTCCGGGCGATGA	TTGCCTTAAACAAGACCACGAAA
Nanog	GCTCCGCTCCATAACTTCGG	TTGTGGGGTGCTAAAATGCG
Fgf5	TACGTGGCCCTGAACAAGAG	TGGAACAGTGACGGTGAAGG
Dnmt3b	GCTTTGCTTCACTGGGTCTC	AACTGATGGGGTACTGACGC
Wnt3	CCTGGACCACATGCACCTAAA	CGGAGGCACTGTCGTACTTG
Dazl	GCAGAGAACCTTTGTACCCAC	TAGCAAGGTGCTGTTCCAGTG
Dnd1	AATGGGTTAAGCAGAGCCTGT	AGGGCAAGGTTCCTCACAAC
Dppa3	CCGTACCTGTGGAGAACAAGA	TAGGGTCTTTCAGCACCGAC
Itgb3	ACGGGACTTTTGAGTGTGGG	GCTGAGAGGGTCGGTAATCC
Nanos3	CACTACGGCCTAGGAGCTTGG	TGATCGCTGACAAGACTGTGG
Prdm1	GAAAAATGGGAGCCCCGACA	AAGACGGAAGGGGACTGTGA
Prdm14	ACAGCCAAGCAATTTGCACTAC	TTACCTGGCATTTTCATTGCTC
Tfap2c	AGGCATCTCATTTCTGCGGG	CACGGACAGGCTTAGAGGTC
Gusβ	GCAGCCCTTCGGGACTTTAT	TCCTCAACACCACTCTCATGTC

Table S3. Lists of DEGs up- or down-regulated in both iPSCs and their corresponding source cell type

Group	DEGs up-regulated in both iPSCs and source cells	DEGs down-regulated in both iPSCs and source cells
**MEFs**	*2510022D24Rik*[1], *Aig1*, *Arhgef2*[2], *Csnk1e*, *Ddx3y*, *Fhl3*, *Fmn1*, *Frmd6*, *Gm10653*, *Gm31458*[3], *Gm8618*, *Hpcal1*, *Klhdc2*, *Lpp*, *Myof*, *Reep1*, *Smim3*, *Zfp800*, *Zswim6*	*1110018N20Rik*[4], *Ahcy*[5], *Cacna1a*, *Ccl25*, *Ccnb1ip1*, *Cdhr1*, *Cep44*, *Commd1*, *Cops7a*, *Crxos*[6], *Dhx38*, *Dnmt3l*, *Dpys*, *Dynlt1a*, *Etv6*, *Fam25c*, *Gadd45gip1*, *Get3*, *Gm10499*, *Gm13778*, *Gm21283*, *Gm40493*, *Gm4737*, *Gm6402*, *Gm8909*, *Gt(ROSA)26Sor*, *Hsd17b14*, *Kbtbd11*, *Lefty1*, *LOC118568154*, *LOC118568782*, *Lsm4*, *Lsm8*, *Mnd1-ps*, *Nek2*, *Nob1*, *Olig1*, *Olig2*, *Pgpep1l*, *Platr26*, *Plbd1*, *Plp2*, *Pmaip1*, *Polr2k*, *Rai1*, *Rfxank*, *Sbds*, *Slc20a2*, *Slc27a2*, *Spire2*, *Taf5l*, *Tm4sf5*, *Tmem17*, *Tomm40*, *Trdmt1*, *Tubb3*, *Ubd*, *Yars2*
**TTFs**	*Arhgef2*, *Arrb1*, *Atp6v0c-ps2*, *Ddx3y*, *Fhl3*, *Fmn1*, *Frmd6*, *Gm31458*, *Gm8618*, *H2ac19*, *Hpcal1*, *Klhdc2*, *Mindy2*, *Myof*, *Nlrx1*, *Pitpnc1*, *S100a13*, *Zfp942*	*1110018N20Rik*, *2210409E12Rik*, *Atp5j*, *Atp6v1b2*, *Cacna1a*, *Car2*, *Cars2*, *Castor1*, *Ccl25*, *Ccnb1ip1*, *Cdhr1*, *Cep44*, *Cherp*, *Cklf*, *Commd1*, *Cops7a*, *Crxos*, *Dnmt3l*, *Dpys*, *Dynlt1a*, *E2f4*, *Fam25c*, *Fbxl14*, *Gadd45gip1*, *Get3*, *Gm10499*, *Gm12338*, *Gm13778*, *Gm22767*, *Gm24289*, *Gm25776*, *Gm31349*, *Gm40847*, *Gm4737*, *Gm5848*, *Gm6360*, *Gm6402*, *Gm8909*, *Gt(ROSA)26Sor*, *Gtpbp3*, *Hsd17b14*, *Isyna1*, *Jam2*, *Kbtbd11*, *Khdc3*, *Klf16*, *Lefty1*, *LOC115488690*, *LOC115490496*, *LOC118568154*,*LOC118568782*, *Lsm4*, *Lsm6*, *Lsm8*, *Miip*, *Msc*, *Mta1*, *Nob1*, *Nop2*, *Nr2c2ap*, *n-R5s111*, *Olig1*, *Olig2*, *Pabpc4*, *Pdf*, *Pet100*, *Pglyrp1*, *Pgpep1l*, *Phb2*, *Pigg*, *Platr26*, *Plp2*, *Pmaip1*, *Polr2k*, *Prr19*, *Raver1*, *Ruvbl2*, *Sbds*, *Serpinb9b*, *Sf3b5*, *Snora33*, *Snora44*, *Spindoc*, *Spire2*, *Srebf1*, *Taf5l*, *Tmem17*, *Tomm40*, *Trdmt1*, *Trmt1*, *Tubb3*, *Ubd*, *Utf1*, *Yars2*, *Zfp869*
**PGCs**	*Atp2b4*, *2510022D24Rik*, *Akap17a*, *Btg1*, *Eif2s3y*, *Fzd3*, *Gm26225*, *Gm31458*, *Gm32885*, *Gm40003*[7], *Hdgfl3*, *Hpcal1*, *Klhdc2*, *Lrrc18*, *Timm8a2*, *Tmem108*, *Ube2q2*, *Uty*, *Xlr3a*, *Xlr3c*, *Xlr4a*, *Xlr4b*	*Ccnb1ip1*, *Crxos*, *Fam25c*, *Fanca*, *Gm10499*, *Gm13778*, *Gm8909*, *Gt(ROSA)26Sor*, *Kbtbd11*, *Lefty1*, *LOC118568782*, *Pcsk9*, *Tmem17*, *Ubd*
**SSCs**	*Egln3*, *Gm10653*, *Gm12643*, *Gm26760*, *Gm40003*, *Timm8a2*, *Tnp1*, *Uty*	*2300002M23Rik*, *Asph*, *Bag3*, *Crxos*, *Dpys*, *Fam25c*, *Gm10499*, *Gm13778*, *Gm6109*, *Gm8909*, *Got2*, *Kbtbd11*, *Lefty1*, *LOC118568782*, *Msc*, *Pdf*, *Tmem17*, *Ubd*

[1] Genes labeled in black are up-regulated DEGs unique in each group.
[2] Genes labeled in blue are up-regulated DEGs common between both MEF and TTF groups.
[3] Genes labeled in orange are up-regulated DEGs common between both MEF, TTF, and PGC groups.
[4] Genes labeled in green are down-regulated DEGs common between both MEF and TTF groups.
[5] Genes labeled in brown are down-regulated DEGs unique in each group.
[6] Genes labeled in red are down-regulated DEGs common in all four groups.
[7] Genes labeled in purple are up-regulated DEGs common between both PGC and SSC groups.

Table S4. Source cell-type specific DEGs detected in iPSCs and transmitted to corresponding PGCLCs

Source cells	Type of DEGs	Potential inheritance of transcriptional memory from source cells detected in iPSCs	Potential inheritance of transcriptional memory from iPSCs detected in PGCLCs
**MEFs**	Up-regulated DEGs	*2510022D24Rik, Aig1, Arhgef2, Csnk1e, Ddx3y, Fhl3, Fmn1, Frmd6, Gm10653, Gm31458, Gm8618, Hpcal1, Klhdc2, Lpp, Myof, Reep1, Smim3, Zfp800, Zswim6*	*Arhgef2*
	Down-regulated DEGs	*Lefty1, Nek2, Trdmt1, Platr26, Gm13778, Slc27a2, Ahcy, 1110018N20Rik, LOC118568782, Sbds, Lsm8, Gt(ROSA)26Sor, Cops7a, Etv6, Plbd1, Crxos, Tomm40, Hsd17b14, Pgpep1l, Ccl25, Kbtbd11, Slc20a2, Gm40493, Cep44, Rfxank, Lsm4, Cacna1a, Gadd45gip1, Get3, Nob1, Dhx38, Gm21283, Spire2, Tubb3, Taf5l, Dnmt3l, Tmem17, Commd1, Rai1, Tm4sf5, Mnd1-ps, Fam25c, Cdhr1, Ccnb1ip1, LOC118568154, Polr2k, Dpys, Yars2, Gm4737, Olig2, Olig1, Dynlt1a, Gm6402, Gm10499, Gm8909, Ubd, Pmaip1, Plp2*	*Dynlt1a*
**TTFs**	Up-regulated DEGs	*Arhgef2, Arrb1, Atp6v0c-ps2, Ddx3y, Fhl3, Fmn1, Frmd6, Gm31458, Gm8618, H2ac19, Hpcal1, Klhdc2, Mindy2, Myof, Nlrx1, Pitpnc1, S100a13, Zfp942*	*S100a13*
	Down-regulated DEGs	*Msc, Gm31349, Lefty1, Trdmt1, Platr26, Gm13778, 1110018N20Rik, Car2, Gm5848, Pabpc4, Snora44, Gm22767, Miip, LOC118568782, Pigg, Sbds, Lsm8, Gt(ROSA)26Sor, Fbxl14, Phb2, LOC115490496, Cops7a, Nop2, Crxos, Pglyrp1, Tomm40, Prr19, Ruvbl2, Hsd17b14, Pgpep1l, Utf1, Pet100, Ccl25, Cars2, Kbtbd11, Cep44, Atp6v1b2, Zfp869, Nr2c2ap, Isyna1, Lsm4, Gtpbp3, Cherp, Gm6360, Lsm6, Cacna1a, Trmt1, Gadd45gip1, Get3, Cklf, E2f4, Pdf, Nob1, Spire2, Tubb3, n-R5s111, Taf5l, Raver1, Gm24289, Khdc3, Sf3b5, Snora33, Dnmt3l, Klf16, Castor1, Tmem17, Commd1, Srebf1, Gm12338, 2210409E12Rik, Gm40847, Gm25776, Mta1, Serpinb9b, Fam25c, Cdhr1, Ccnb1ip1, LOC118568154, Polr2k, Dpys, Yars2, LOC115488690, Gm4737, Jam2, Atp5j, Olig2, Olig1, Dynlt1a, Gm6402, Gm10499, Gm8909, Ubd, Pmaip1, Spindoc, Plp2*	*Dynlt1a, Gadd45gip1, Gm12338, Khdc3, Lsm8, Pmaip1, Prr19, Taf5l*
**PGCs**	Up-regulated DEGs	*Atp2b4, Gm40003, Hdgfl3, Ube2q2, Tmem108, Btg1, Hpcal1, Klhdc2, Lrrc18, Gm26225, Fzd3, Timm8a2, Gm31458, Gm32885, Xlr4a, Xlr3a, Xlr4b, Xlr3c, 2510022D24Rik, Akap17a, Eif2s3y, Uty*	N/A*
	Down-regulated DEGs	*Ccnb1ip1, Crxos, Fam25c, Fanca, Gm10499, Gm13778, Gm8909, Gt(ROSA)26Sor, Kbtbd11, Lefty1, LOC118568782, Pcsk9, Tmem17, Ubd*	N/A
**SSCs**	Up-regulated DEGs	*Egln3, Gm10653, Gm12643, Gm26760, Gm40003, Timm8a2, Tnp1, Uty*	*Egln3*

	Down-regulated DEGs	*2300002M23Rik, Asph, Bag3, Crxos, Dpys, Fam25c, Gm10499, Gm13778, Gm6109, Gm8909, Got2, Kbtbd11, Lefty1, LOC118568782, Msc, Pdf, Tmem17, Ubd*	N/A

*No genes found.
